# E-cadherin focuses protrusion formation at the front of migrating cells by impeding actin flow

**DOI:** 10.1038/s41467-020-19114-z

**Published:** 2020-10-26

**Authors:** Cecilia Grimaldi, Isabel Schumacher, Aleix Boquet-Pujadas, Katsiaryna Tarbashevich, Bart Eduard Vos, Jan Bandemer, Jan Schick, Anne Aalto, Jean-Christophe Olivo-Marin, Timo Betz, Erez Raz

**Affiliations:** 1 Institute of Cell Biology, Center for Molecular Biology of Inflammation, University of Münster, 48149 Münster, Germany; 2 Institut Pasteur, Bioimage Analysis Unit, 75105 Paris, France; 3 CNRS UMR 3691, 75105 Paris, France; 4 Sorbonne Université, 75005 Paris, France; 5 Present Address: Institute of Physics − Biophysics, Georg August Universität, Friedrich-Hund-Platz 1, 37077 Göttingen, Germany

**Keywords:** Adherens junctions, Cell migration, Actin, Embryology

## Abstract

The migration of many cell types relies on the formation of actomyosin-dependent protrusions called blebs, but the mechanisms responsible for focusing this kind of protrusive activity to the cell front are largely unknown. Here, we employ zebrafish primordial germ cells (PGCs) as a model to study the role of cell-cell adhesion in bleb-driven single-cell migration in vivo. Utilizing a range of genetic, reverse genetic and mathematical tools, we define a previously unknown role for E-cadherin in confining bleb-type protrusions to the leading edge of the cell. We show that E-cadherin-mediated frictional forces impede the backwards flow of actomyosin-rich structures that define the domain where protrusions are preferentially generated. In this way, E-cadherin confines the bleb-forming region to a restricted area at the cell front and reinforces the front-rear axis of migrating cells. Accordingly, when E-cadherin activity is reduced, the bleb-forming area expands, thus compromising the directional persistence of the cells.

## Abstract (web summary)

The arrival of migratory cells at their targets relies on following precise routes within tissues. Here the authors demonstrate that the cell adhesion molecule E-cadherin can control the path of cell migration by confining the site where bleb-type protrusions form within the cell front.

## Introduction

For a variety of cell types in physiological and disease contexts, cell motility is powered by actomyosin-dependent protrusions called blebs^1–4^. The site of bleb initiation^4^ has been suggested to be governed by various molecular and biophysical processes, including intracellular events such as myosin contraction^5–7^, local weakening of the cortex and its attachment to the cell membrane^8–11^ and the generation of areas of negative membrane curvature by previous blebs or pseudopods^12^. By employing these cell-autonomous mechanisms, cells can preferentially produce blebs on one side of the cell, thereby defining a leading edge and promoting migration. Importantly, unlike the “stable bleb” phenomenon that is readily induced by strong experimental enhancement of cortical contractility^6,7^ and unlike situations where bleb-driven movement is physically confined^13^, migration of blebbing cells characteristically involves the formation of multiple transient protrusions at different locations. Thus, in the context of single-cell migration in vivo, coordinating blebbing activity within the leading edge is critical for dictating the precise migration direction. However, the molecular mechanisms responsible for focusing this kind of protrusive activity to a limited part of a migrating cell’s front are not well defined. In particular, the precise role played by the interaction between migratory cells and cells in their environment in controlling the polarized formation of blebs is currently unknown. An excellent model for studying these issues in the context of the live organism is that of the primordial germ cells (PGCs), cells that migrate within embryos of different organisms to reach the region where the gonad develops^14,15^.

In this study, utilizing a range of genetic, reverse genetic and mathematical tools, we reveal a role for E-cadherin in confining bleb-type protrusions to the leading edge of migrating zebrafish PGCs. We show that polarized PGCs present actin filaments at the cell front that, in turn, can recruit myosin molecules. This actomyosin enrichment generates a gradient of contractility that biases the formation of blebs at the leading edge. In a wild-type scenario, the coupling of actin in PGCs to that in surrounding cells via E-cadherin impedes the backwards flow of actomyosin-rich structures so that these structures remain confined to the leading edge. In this way, E-cadherin limits the bleb-forming region to a restricted area of the cell and reinforces the front-rear axis of migrating cells. Accordingly, upon depletion of E-cadherin, the bleb-forming area expands and bleb formation is less focused, thus compromising the directional persistence of the migrating PGCs and their arrival at the target.

## Results

### E-cadherin influences the straightness of cell migration tracks

**Fig. 1:**
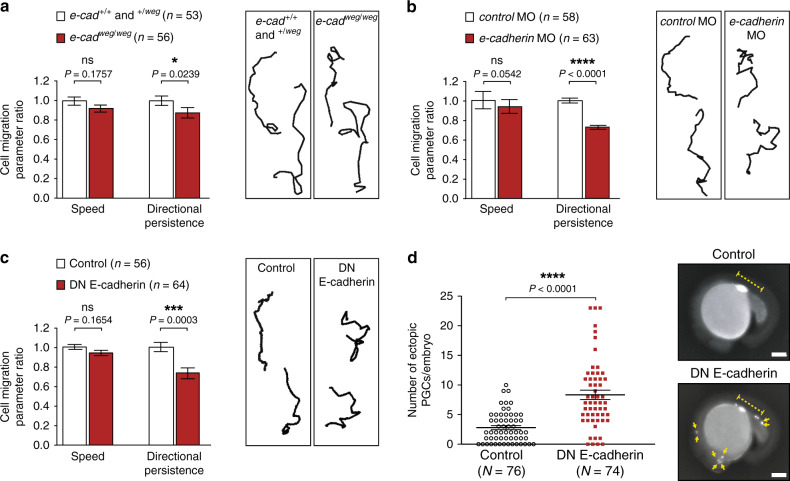
E-cadherin is required for germ cell directionally persistent migration track. **a** Graph: PGC migration speed and directional persistence in *e-cadherin*^*weg/weg*^ embryos relative to control embryos. *n* = number of cells from 4 independent experiments. Right: representative tracks of PGCs migrating within *e-cadherin*^*+*/*+*^ and ^+/*weg*^ embryos or *e-cadherin*^*weg/weg*^ embryos. **b** Graph: PGC migration speed and directional persistence in *e-cadherin*-morpholino-treated embryos relative to control embryos. *n* = number of cells from 4 independent experiments. Right: representative tracks of PGCs migrating within embryos injected with either a *control* or *e-cadherin* morpholino. **c** Graph: migration speed and directional persistence of PGCs expressing a dominant-negative form of E-cadherin as compared with control cells. *n* = number of cells from 3 independent experiments. Right: representative tracks of cells expressing a control protein or a dominant-negative form of E-cadherin. The results presented in **a**–**c** were derived from time-lapse movies captured between 6 and 8 hours post fertilization (hpf) at a time interval of 2 min between frames. The tracks are presented as 2-dimensional Z-projection of the 3-dimensional data acquired. Cells were tracked for 70 min and analyzed using Imaris software. Additional representative tracks are provided in Supplementary Fig. 1a. Normalized mean ± s.e.m.; *P* value: two-tailed Mann–Whitney *U*-test; ns = not significant. **d** Graph: number of cells that reside out of the developing gonad region at 22 hpf. Mean ± s.e.m.; *P* value: two-tailed Mann–Whitney *U-*test; *N* = number of embryos from 3 independent experiments. Right: Dashed yellow line indicates the developing gonad region, which spans the first half of the yolk extension; yellow arrows point at ectopic cells; scale bars, 150 μm. Source data are provided as a Source Data file.

Our previous work on bleb-driven cell migration showed that when placed within a cell-free three-dimensional extracellular matrix environment, zebrafish PGCs exhibited an extensive and apolar formation of blebs^16^; these results suggested that the migration of PGCs requires interactions with cells in their environment. To assess the role of cell–cell adhesion in bleb-driven single-cell migration, we examined the function of E-cadherin, which is expressed in early zebrafish embryos^17,18^. We first characterized different cell migration parameters in *weg*^*tx230*^ mutant embryos, which carry a point mutation in the *e-cadherin* gene resulting in a non-functional version of the protein^17^. Strikingly, PGCs migrating within *e-cadherin*^*weg/weg*^ mutant embryos did not show a significant reduction in their migration speed, but the straightness of their migration path was affected (Fig. 1a and Supplementary Fig. 1a). Interestingly, although E-cadherin appeared to sustain the directional migration course of PGCs, its overall distribution around the cell perimeter was not polarized (Supplementary Fig. 2). Since in the zebrafish both E-cadherin RNA and protein are maternally provided to the embryo^17,18^, the loss of zygotically transcribed *e-cadherin* represents only a partial loss of function concerning PGC migration and early embryonic development (homozygous *e-cadherin*^*weg/weg*^ mutant adults cannot be generated, as the mutation is lethal). Accordingly, the level of E-cadherin in *e-cadherin*^*weg/weg*^ embryos was not dramatically different from that in the wild-type and *e-cadherin*^*+/weg*^ siblings (Supplementary Fig. 1b), which had to be pooled together as the *e-cadherin*^*+/weg*^ embryos do not show a somatic phenotype. In contrast, when inhibiting the translation of both maternally provided and zygotically transcribed *e-cadherin* RNA using specific morpholino (MO) antisense oligonucleotides^18,19^ (Supplementary Fig. 1c, e), the protein levels were strongly reduced (Supplementary Fig. 1d). This manipulation, which reduced the levels of the adhesion molecule in PGCs and in the cells surrounding them, strongly affected the migration path, which became more convoluted (Fig. 1b and Supplementary Fig. 1a). However, similar to the findings in the zygotic mutant, the average cell migration speed was not significantly altered (Fig. 1b).

Next, to also inhibit the function of the maternally provided E-cadherin protein, we expressed in the embryos a version of E-cadherin that harbours the point mutation W2A (Tryptophan 2 to Alanine, hereafter DN E-cadherin). The mutated protein acts as a dominant-negative version by interfering with E-cadherin–E-cadherin trans interactions^20,21^, and was localized to the membrane in both somatic cells and PGCs (Supplementary Fig. 1f). Importantly, when expressed in all the cells of the embryo, DN E-cadherin induced the expected effects on gastrulation progression and epiboly movements^17^ (Supplementary Fig. 1g), demonstrating that it can interfere with the function of the endogenous E-cadherin molecules. Similar to the results obtained upon global knockdown of E-cadherin using a genetic mutation or MO (Fig. 1a, b), the expression of DN E-cadherin specifically within the PGCs themselves strongly decreased the directional persistence of the migration without affecting the cell speed (Fig. 1c and Supplementary Fig. 1a). As a result of the impaired migration course, a high proportion of the manipulated cells failed to colonize the gonad region at 22 hours post fertilization (hpf) as compared with control cells (Fig. 1d). Consistent with the results presented above, ectopic localization was also observed for PGCs expressing a mutant version of β-catenin that interferes with the function of E-cadherin^22^ (Supplementary Fig. 3).

Comparing our results with those presented before^16^, we found that reducing E-cadherin function can indeed lead to an increase in cases where cells show very little displacement (Fig. 1a–c and Supplementary Fig. 1a), especially when the E-cadherin function is knocked down using morpholinos that affect the environment as well. Importantly, however, differently from previous conclusions^16^, upon measuring the average migration speed of a large number of cells, we observed only a mild and non-statistically significant reduction (Fig. 1a–c). We attribute the discrepancy to the fact that, now, a much larger number of cells can be monitored using more advanced and higher-throughput imaging equipment (e.g., spinning-disk confocal microscopes employing motorized stages), and that the use of state-of-the-art software allows for more reliable analysis of the data (e.g., unbiased tissue drift correction).

Together, our results show that E-cadherin-mediated cell–cell adhesion is primarily required for ensuring a straight path of migration rather than for PGC motility per se.

### Control of actin distribution in the cell by E-cadherin

**Fig. 2:**
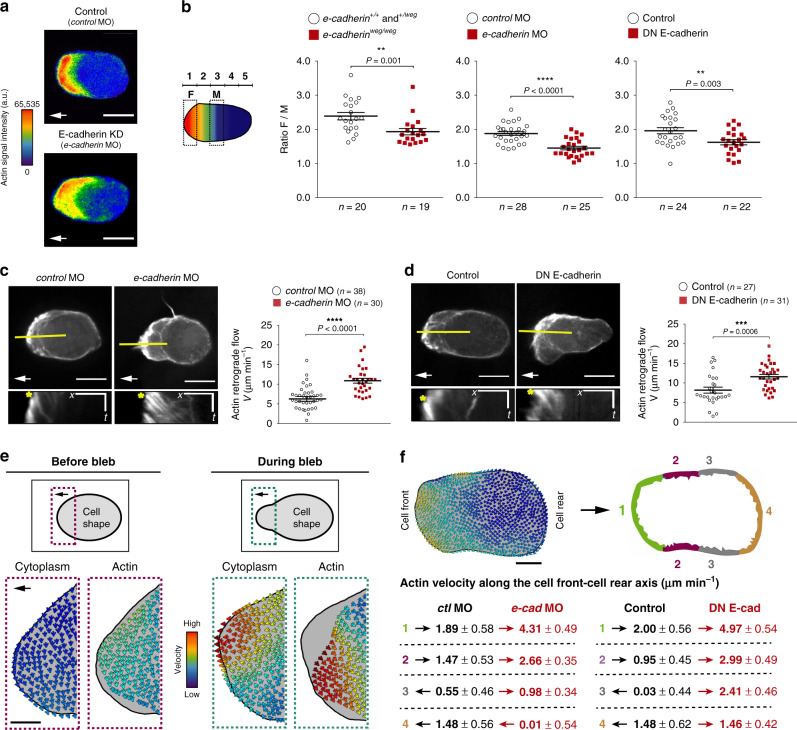
Effect of E-cadherin depletion on actin distribution and dynamics within migrating PGCs. **a** Color-coded actin (LifeAct-EGFP) fluorescence intensity in a control cell (upper panel) and in an E-cadherin-depleted cell (lower panel). White arrows indicate the migration direction; scale bars, 10 μm. Cells are derived from the same data sets presented in **b** and in Supplementary Fig. 4a, b. **b** Left panel: schematic illustration of the division of the cell into 5 domains employed in the quantitation presented on the right. The ratio between the mean value of LifeAct-EGFP signal in segment 1 (F, front) and that in segment 3 (M, middle) was calculated. Graphs: F/M ratios for control cells and E-cadherin-depleted cells (*e-cadherin*^*weg/weg*^ mutants (left graph), *e-cadherin* morpholino-injected embryos (middle graph), and DN E-cadherin-expressing PGCs (right graph)). *n* = number of cells from 5 independent experiments for *e-cadherin*^*weg/weg*^ mutants and 4 independent experiments for the other conditions; mean ± s.e.m.; *P* value: two-tailed Mann–Whitney *U-*test. Data derived from the same sets of cells analyzed in Supplementary Fig. 4a, b. **c**, **d** Upper panels: representative polarized PGCs (control cells and cells treated with either *e-cadherin* morpholino (**c**) or DN E-cadherin (**d**)) expressing LifeAct-EGFP. White arrows indicate the direction of migration; scale bars, 10 μm. Lower panels: kymographs along the yellow lines in the upper panels. Yellow stars mark the starting position on the cell border where kymograph measurements were conducted (see Methods for further details). Scale bars, *x* = 5 μm, *t* = 12 s (s = seconds). Graphs: actin velocity values at the cell front derived from the kymographs. *n* = number of velocities from 8 independent experiments for morphants (**c**) and 6 independent experiments for DN E-cadherin (**d**); mean ± s.e.m.; *P* value: two-tailed Mann–Whitney *U*-test. **e** Upper schematics: representative shape of a polarized PGC before (left) and after (right) bleb initiation. Black arrows indicate the direction of migration. Dotted rectangles mark the regions of the cell front, which are magnified in the panels below. Lower panels: Color-coded arrows represent the direction of flow of cytoplasm (cytosolic EGFP) and actin (LifeAct-mCherry) at the cell front of a representative migrating PGC before (left, purple panels) and during bleb (right, green panels). For both channels, the arrows indicating the flow are placed on top of the cell contour derived by edge detection for the cytoplasmic signal (gray background). The partial snapshots are derived from the time points 6.0 s and 12.5 s in the Supplementary Movie 2. Cytoplasmic EGFP: red = 44.4 μm min^−1^; blue = 0.0 μm min^−1^. LifeAct-mCherry: red = 46.8 μm min^−1^; blue = 0.0 μm min^−1^. Black arrow indicates the direction of migration; scale bar, 5 μm. **f** Measurements of actin velocity along the cell perimeter performed using the BioFlow software (see Methods). Snapshot: the length of the cell contour was divided into four equal parts with an average width of 1.7 μm. The mean actin velocity values along the cell front-rear axis within these regions were calculated for 10 s prior to bleb formation. The presented snapshot corresponds to an example control cell: red = 28.2 μm min^−1^; blue = 0.0 μm min^−1^. Scale bar = 5 μm. Tables: black and red arrows indicate the direction of actin movement with respect to the cell front-rear axis. Values indicate mean ± s.e.m. Data are derived from the same sets of cells analyzed in **c**, **d**. Source data are provided as a Source Data file.

For cells to achieve directional migration, they must maintain cell polarization along their front-rear axis^23^. The cell front of migrating PGCs contains actin-rich structures at the base of forming blebs (Fig. 2a, and ref. ^5^). Since depleting E-cadherin reduced the directional persistence of PGC migration (Fig. 1a–c and Supplementary Fig. 1a) and since the cytoplasmic domain of E-cadherin can be linked to actin by proteins of the catenin family^20^, we examined whether depleting E-cadherin affects the distribution of actin in motile PGCs. Upon the reduction of E-cadherin function using each of the tools presented above, the enrichment of actin at the cell front decreased relative to that in the rest of the cell body (Fig. 2a, b and Supplementary Fig. 4a) without affecting the total level of actin in the cell (Supplementary Fig. 4b), indicating that E-cadherin functions in maintaining actin polarization. To gain further insight into the mechanism by which E-cadherin sustains actin polarization, we disrupted its interaction with actin by expressing the mutant form of β-catenin that lacks the binding site for α-catenin, a treatment that affected PGC migration (Supplementary Fig. 3). Similar to the findings obtained when reducing E-cadherin function (Fig. 2a, b and Supplementary Fig. 4a, b), this manipulation interfered with the polar distribution of actin within the cells (Supplementary Fig. 4c–e).

We have previously shown that the enrichment of actin at the leading edge of polarized PGCs depends on the persistent elevated activity of the Rho GTPase Rac1 at this part of the cell^16^. Since E-cadherin functions in a positive feedback loop with Rac1 to stabilize forward-directed protrusion and promote directionally persistent movement in the context of collective cell migration^24^, we tested the hypothesis that E-cadherin acts similarly in migrating PGCs. For that purpose, we measured the activation of Rac1 in the PGCs upon E-cadherin depletion using a FRET-based sensor^16,25^. E-cadherin knockdown did not affect Rac1 activity neither globally in the whole cell nor specifically at the leading edge (Supplementary Fig. 5a, b), indicating that cell–cell adhesion supports PGC polarity via an alternative mechanism. In addition, we had previously shown that PGCs are guided in their migration by the chemokine Cxcl12a^26^, which activates signalling cascades that lead to Rac1 activation at the leading edge^27^. Since Rac1 activity remained unchanged upon E-cadherin depletion, it is unlikely that reduced levels of cell–cell adhesion affect PGC polarity and directional migration by altering their perception of Cxcl12a.

Previous studies have shown that E-cadherin can control actomyosin flows in different contexts^28,29^, and that actin flows can play a role in the establishment and maintenance of cell polarity in migration^30–34^. We thus investigated the possibility that E-cadherin controls PGC polarity by affecting actin distribution in the cells. To address this issue, we measured the velocity of actin in polarized cells upon knockdown of E-cadherin. In these and the following experiments, we utilized either *e-cadherin* MO or DN E-cadherin rather than *e-cadherin*^*weg/weg*^ embryos, which exhibited similar albeit weaker phenotypes (Fig. 1) due to the presence of maternally provided RNA and protein (compare Supplementary Fig. 1b, d). Interestingly, measurements of actin dynamics at the leading edge of control cells confirmed the presence of retrograde flow, and this appeared to be strongly increased both upon global E-cadherin depletion (employing the *e-cadherin* MO, Fig. 2c and Supplementary Movie 1) and when E-cadherin function was specifically inhibited in the PGCs (DN E-cadherin, Fig. 2d and Supplementary Movie 1).

Since kymographs can provide information regarding the velocity of the flow only at one arbitrarily defined point, we set out to assess whether reducing E-cadherin would affect the dynamics of actin around the entire cell contour. To this end, we employed the BioFlow software, which allows for non-invasive, global visualization and analysis of flow fields based on temporal analysis of the positioning of fluorescently labeled structures^35^. In our study, as it has been described previously^36^, the BioFlow software reported a movement of cytoplasmic EGFP into the forming bleb; however, we also found that the mCherry-labeled actin structures were flowing away from the region of the forming protrusion (Fig. 2e and Supplementary Movie 2). In addition, upon a similar analysis conducted prior to bleb formation, we also observed retrograde flow of the E-cadherin-EGFP molecule, although at a slower speed than that of actin (Supplementary Fig. 5c). This observation supports the idea that E-cadherin and a fraction of actin physically interact, as suggested for other systems^28,37^.

We then tested the ability of the software to detect changes in the velocity of the actin flow by performing measurements in cells where the activity of RhoA was increased by expressing the constitutively active version V14RhoA (caRhoA)^38^. In cells expressing the deregulated version of RhoA, enhanced retrograde flow in one direction is further amplified by depletion of actin and myosin from one aspect of the cell^6,16^. As a consequence, the contractility machinery is transported to one side of the cell leading to persistent cell polarization. Similarly, the software reported a strongly enhanced retrograde flow of actin at the cell front of PGCs that express caRhoA (Supplementary Fig. 5d). Consistent with the idea that the retrograde flow depends mainly on myosin activity, overexpression of a dominant-negative form of the Rho kinase protein^5,39^ (DN-ROCK) strongly inhibited the backwards movements of actin (Supplementary Fig. 5e). Interestingly, when we applied the software to analyse the actin flow around the entire cell contour, it revealed that downregulating E-cadherin led to significantly higher velocities of actin retrograde flow at the cell front and along the cell sides. Within these regions in wild-type cells the retrograde flow would usually slow down and eventually revert (Fig. 2f and Supplementary Movie 3). Faster actin retrograde flow was also measured when the interaction between E-cadherin and actin was inhibited by expressing a mutant version of β-catenin (Supplementary Fig. 5f).

Together, our observations show that in PGCs, myosin-dependent contractility generates a global retrograde movement of E-cadherin and actin, including the actin-rich structures present at the cell front. Moreover, our results are consistent with the idea that E-cadherin couples the cortical actin in the germ cells to that in neighbouring cells, thereby increasing friction and reducing the backward flow of actin. Accordingly, when E-cadherin was knocked down, which lowered the friction component, we observed more robust retrograde actin flow. Thus, E-cadherin may support actin polarization by limiting its backwards movement. Indeed, inhibiting myosin-based contraction (and therefore actin flow (Supplementary Fig. 5e)), abrogated the changes in actin distribution caused by the depletion of E-cadherin (compare Supplementary Fig. 6a, c to Fig. 2a, b and Supplementary Fig. 6b to Supplementary Fig. 4a).

### Bleb distribution at the cell front is regulated by E-cadherin

Our findings show that E-cadherin knockdown results in a faster retrograde flow of actin, which is correlated with more convoluted migration paths. Interestingly, other studies have shown that increasing the speed of actin retrograde flow can increase cell polarity, thereby promoting directional migration persistence^6,7,30,40^. To address this discrepancy, we examined in more detail the basis for the reduction in directionality that we observed upon increasing actin flow, focusing on the generation of blebs at the leading edge.

**Fig. 3:**
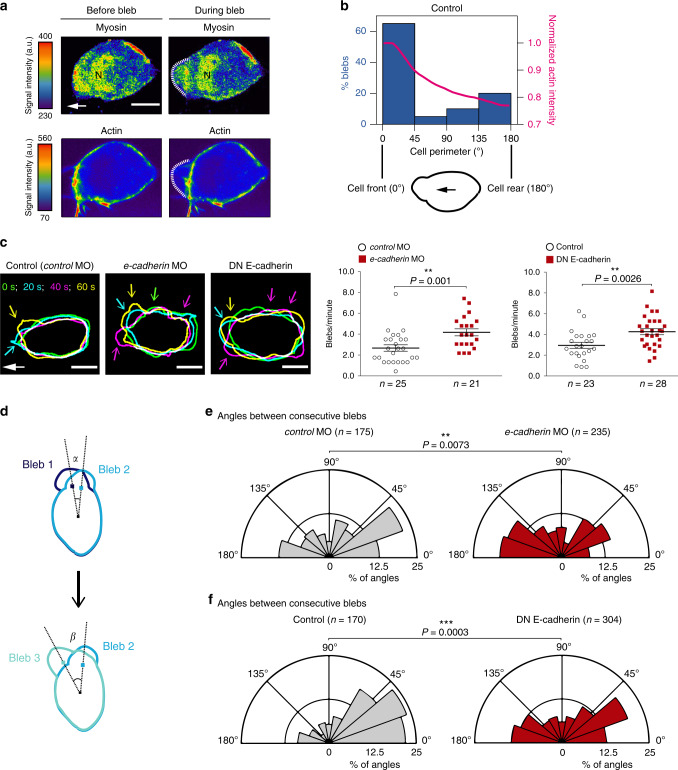
Myosin distribution in migrating PGCs and changes in the polarized generation of blebs upon E-cadherin knockdown. **a** Snapshots showing the distribution of myosin (Myl12.1-EGFP) and actin (LifeAct-mCherry) in a polarized PGC before and during bleb formation (see Supplementary Fig. 7a for an additional example). The snapshots are derived from Supplementary Movie 4, cell1, time points 0 s (before bleb) and 8 s (during bleb). White arrow indicates the direction of migration; *N* = myosin nuclear localization that might reflect a possible function of the protein in the nucleus (as described for human Myl12a also known as MRLC3^62^); a.u. = arbitrary units; scale bars, 10 μm. The experiment was repeated three times. **b** Graph: percentage of blebs initiating at different angles around the cell perimeter (left *y*-axis, blue columns) and normalized actin intensity (right *y*-axis, magenta line) of Control cells (for cells expressing the DN E-cadherin and analyzed in a similar way see Supplementary Fig. 8c). The schematics shows the cell perimeter of a polarized PGC where 0° represents the cell front and 180° the cell rear. The black arrow indicates the direction of migration. A total number of 4 representative cells and 20 blebs were analyzed. **c** Left panels: overlays of the cell contours of three representative polarized PGCs at four time points, presenting bleb formation over 60 s. Time points are color-coded as indicated. Colored arrows point at blebs formed at time points of the corresponding colors. White arrow indicates the direction of migration; scale bars, 10 μm; s = seconds. The cell contours are derived from the movies in Supplementary Movie 6 (*control* MO: 0, 20, 40, 60 s; *e-cadherin* MO: 20, 40, 60, 80 s; DN E-cadherin: 15, 35, 55, 75 s). Graphs present the number of blebs per minute in polarized, motile PGCs. *n* = number of cells from 6 and 5 independent experiments for *e-cadherin* morpholino experiments and DN E-cadherin experiments, respectively; mean ± s.e.m.; *P* value: two-tailed Mann–Whitney *U*-test for morpholino and two-sided Student’s *t*-test for DN E-cadherin. **d** Schematics explaining the measurements provided in **e** and **f**. The angles between consecutive blebs were derived by measuring the angle between the two lines that connect the centre of the cell with the point of bleb initiation at a certain time point (for example, time point 1, bleb 1) and the site of initiation of the next bleb (for example, time point 2, bleb 2). **e**, **f** Polar plots show the distribution of angles between consecutive blebs in control cells (gray polar plots) and cells with manipulated E-cadherin function (red polar plots). *n* = number of angles obtained from 7 independent repeats for morpholino experiments (26 cells for *control* MO and 24 cells for *e-cadherin* MO) and 6 independent repeats for the DN E-cadherin experiments (23 cells for Control and 31 cells for DN E-cadherin). The time-lapse videos for characterizing blebbing were captured at 500 ms time intervals. *P* values: two-tailed Kolmogorov–Smirnov test. Source data are provided as a Source Data file.

Bleb initiation depends on actomyosin contractility, which can induce local ruptures in the actin cortex or lead to the local detachment of the cortex from the plasma membrane^4,41^. In polarized PGCs, elevated levels of actin are found at the cell front (Fig. 2a). As actin and myosin interact with one another^42^, the enrichment of actin at the leading edge could lead to an increased imbalance in the contractile forces generated at this location. As a consequence, the actin cortex could preferentially tear there, resulting in a biased formation of blebs at the cell front. Consistent with this possibility, polarized PGCs show elevated myosin activity at their leading edge and at the site of bleb formation, as reflected by the localization of the Myosin regulatory light chain 12.1 (Myl12.1) (Fig. 3a, Supplementary Fig. 7a, and Supplementary Movie 4). The high correlation between the site of bleb formation and actin enrichment could be demonstrated by simultaneously determining their relative position around the cell perimeter (Fig. 3b). The instructive role of actin in biasing the position where blebs form was further proven by impairing its polar distribution using uniform high expression of the guidance cue Cxcl12a^26^. This manipulation resulted in the formation of blebs around the entire cell circumference rather than at a specific aspect of the cell (Supplementary Fig. 7b and Supplementary Movie 5).

Based on these results, it is expected that the change in actin distribution observed upon E-cadherin knockdown (Fig. 2a, b and Supplementary Fig. 4a) would be accompanied by a parallel change in myosin polarization, thereby affecting the position of bleb formation. To test this hypothesis, we first quantified the frequency of bleb formation; in cells depleted of E-cadherin, this frequency increased (Fig. 3c). Similar results were obtained by expressing in the cells the mutant version of β-catenin that inhibited the interaction between E-cadherin and actin (Supplementary Fig. 8a). Judging by the fact that the global level of RhoA activation was not altered in the treated cells (Supplementary Fig. 8b), the basis for the enhanced blebbing activity was not a general increase in contractility.

Strikingly, reducing E-cadherin function not only increased the frequency of blebbing events but also interfered with the focused formation of blebs at the leading edge of the cell (Fig. 3c, left panels, Supplementary Movie 6 and Supplementary Fig. 8c). To quantify this effect, we measured the angle between consecutive protrusions as illustrated in Fig. 3d. In control cells, the majority of sequential blebs occurred close to each other, with most of the angles falling in the range of 0°–40° (Fig. 3e, f, gray polar plots on the left). The very large angles (160°–180°) between consecutive blebs are likely to result from protrusions initiated by the elevated levels of myosin at the rear of polarized cells (Fig. 3a and Supplementary Movie 4), which is known to facilitate back retraction^5^. Interestingly, upon reducing either the amount or the function of E-cadherin, the distribution of consecutive blebs was strongly affected, leading to a corresponding change in cell shape. Specifically, the percentage of consecutive blebs forming closer to one another sharply decreased compared to control cells, while the proportion of sequential blebs forming at broader angles significantly increased (Fig. 3e, f, compare red polar plots to the corresponding control gray polar plots). Interestingly, disrupting the link between E-cadherin and actin via the expression of a β-catenin mutant also reduced the percentage of consecutive blebs forming close to each other (Supplementary Fig. 8d). These phenotypes did not result from a change in contractility at the cell front, as the level of RhoA activation at the leading edge relative to that at other parts of the cell was not altered in cells with compromised E-cadherin function (Supplementary Fig. 8e).

Together, these data clearly show that the interaction between E-cadherin and actin plays an important role in confining the formation of blebs to a defined region of the cell, thereby promoting persistent direct migration paths.

### A model for regulating bleb distribution at the cell front by adhesion

**Fig. 4:**
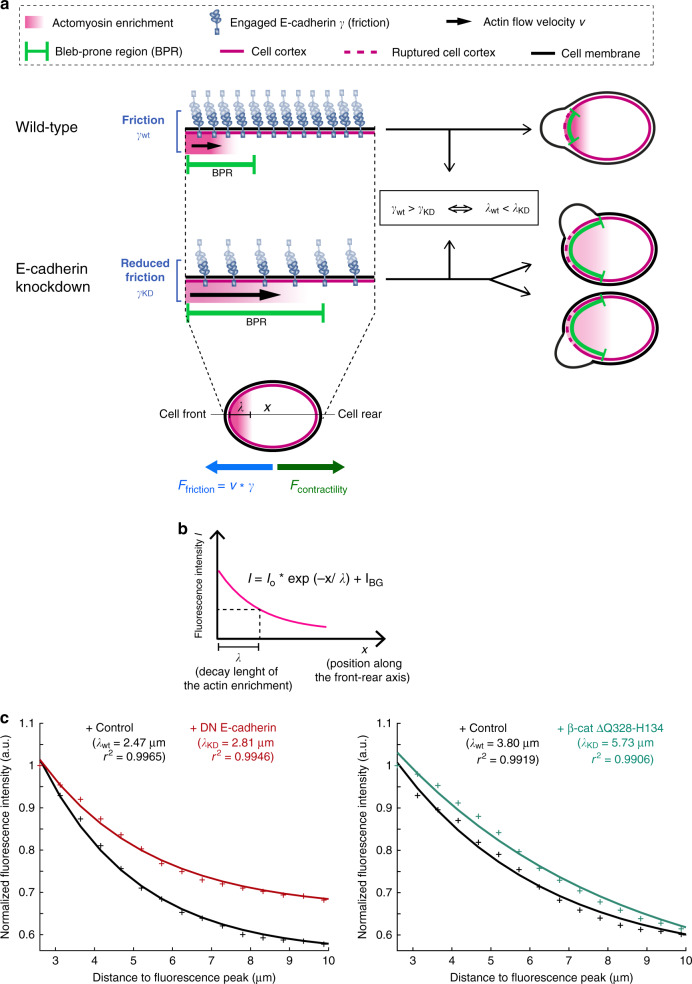
A model for the function of E-cadherin in sustaining polarized formation of protrusions in bleb-based migration. **a**, **b** Actomyosin enrichment (magenta gradient) at the front of migrating cells promotes bleb initiation either by causing breaks within the actin cortex or by detaching the cortex from the plasma membrane. Accordingly, the region of the cell front containing a high level of actomyosin is more prone to subsequent blebbing (bleb-prone region, BPR). Myosin-dependent contractility also generates a contractile force (*F*_contractility_) that results in retrograde cortical flow, together with the actomyosin-rich structure present at the cell front (Actin flow velocity *v*, black arrow). In wild-type cells, actin in PGCs is coupled to that in surrounding cells via E-cadherin; this coupling restricts the bleb-prone region at the leading edge by frictional forces resisting the actomyosin-driven flows (*γ*_wt_, wild-type). Upon a depletion of E-cadherin (E-cadherin knockdown scenario), the friction opposing the actin retrograde flows (*γ*_KD_) is lower. As a result, the bleb-prone region spreads towards the back of the cell, and blebbing events occur in a less coordinated manner. The shape of the spread can be predicted by a transport-decay model to be an exponential function, where *λ* is a characteristic length that depends inversely on the friction *γ*, and *I*_BG_ is the background fluorescence. (For more details on the mathematical modelling, see Methods). **c** Exponential fits of the actin fluorescence intensity in the PGCs derived from the signal profiles shown in Supplementary Fig. 4a, d upon decreasing the actin to membrane linkage by the expression of DN E-cadherin or β-catenin mutant (for the fits of *e-cadherin*^*weg/weg*^ mutant and *e-cadherin* MO see Supplementary Fig. 9). As predicted by the transport-decay model, in each case the decay length of E-cadherin knockdown (*λ*_KD_) is larger than in the control (*λ*_wt_) situation: *λ*_KD_ > *λ*_wt_. This suggests that the overall actin polarity is reduced when the friction with the environment is reduced.

Based on the experimental results presented so far, we propose a role for cell–cell adhesion in enhancing cell polarity during bleb-driven migration, as depicted schematically in Fig. 4a. An essential requirement for persistent single-cell migration along straight paths is the focused formation of protrusions in the direction of movement. Accordingly, for unpolarized blebbing cells to become motile, they must convert transient fluctuations in cell contractility or in membrane-to-cortex attachment into temporally persistent asymmetries. A way of doing that is to generate stable contractility gradients that would favor bleb formation at a specific region around the cell perimeter (named “Bleb-prone region” or “BPR”, green in Fig. 4a).

Polarized PGCs can generate such a gradient by assembling actin filaments at the cell front that, in turn, could recruit myosin molecules (indicated as “Actomyosin enrichment”, magenta in Fig. 4a). By exerting stress on the actin cortex, this actomyosin enrichment initiates the mechanisms required for bleb formation; namely, it creates ruptures in the cell cortex or causes the cortex to detach from the plasma membrane^4^. Importantly, at the same time, myosin-dependent contractility generates a force (*F*_contractility_) that leads to a retrograde flow of the cortical actomyosin together with the actomyosin-rich structure present at the cell front (Fig. 4a, retrograde velocity defined as *v*). In the wild-type scenario, the coupling of actin in PGCs to that in surrounding cells via E-cadherin limits the backwards flow of actomyosin by an effective friction (*γ*_wt_), and, consequently, restricts the spread of the bleb-prone region at the leading edge (Fig. 4a, wild-type). Depleting E-cadherin lowers the friction that impedes the actin retrograde flows (*γ*_KD_), thereby extending the spread of the bleb-prone region towards the sides of the cell (Fig. 4a, E-cadherin knockdown). As a result, upon depletion of E-cadherin, bleb formation is less focused, compromising the cells’ ability to directionally migrate towards their target.

Overall, we propose that E-cadherin enhances the polarization of actomyosin along the front-rear axis, thereby focusing the formation of blebs at the front of the migrating cells.

To examine this schematic model, we first developed a theoretical description of the measured actin distribution in the PGCs and then compared it with experimental results. The actin polymerization rate is high at the cell front and is then greatly reduced away from this region as retrograde flow leads to actin filaments streaming towards the cell rear. This effective reduction of the polymerization component leads to a net actin network disassembly at a constant depolymerization rate, as actin flows away from the cell front. This transport-degradation approach predicts an exponential decay of the actin fluorescence signal in the transport region (Fig. 4b), which is indeed reflected by the very low variance between the experimentally measured values and the fitted curves (mean *r*² = 0.993) (Fig. 4c, Supplementary Fig. 9, and Methods). In this theoretical description, the experimentally determined decay length *λ* depends inversely on the friction (*λ* ∝ *γ*^−1^). Accordingly, an increase in *λ* is expected upon a reduction in friction, for example as a result of inhibiting the function of E-cadherin or β-catenin. Indeed, we observe an increase in *λ* upon disruption of the actin linkage in cells in which E-cadherin or β-catenin function was compromised as compared to control cells (Fig. 4c and Supplementary Fig. 9). Thus, a balance between friction, contraction and depolymerization can quantitatively account for the actin distribution as previously shown in other systems^7,43^. Together, the increased retrograde flow due to a reduction in E-cadherin function can increase *λ*, which reduces the polarity of migrating PGCs (Fig. 4a, b).

### The adhesion level within the environment affects the migration path

**Fig. 5:**
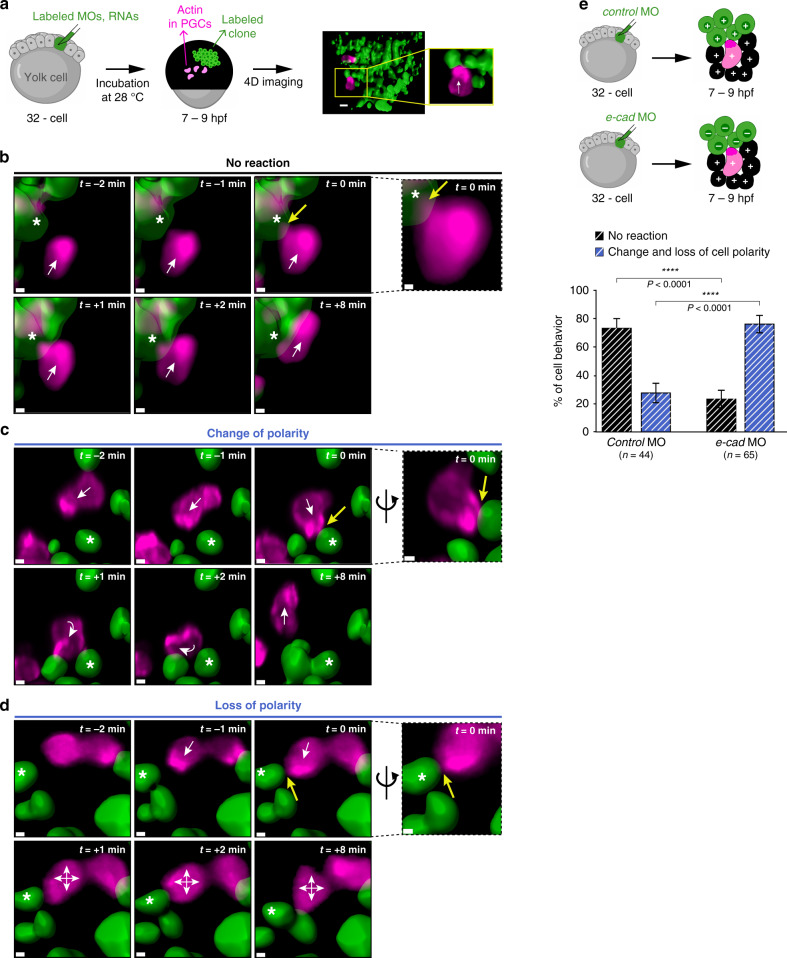
Behavior of migrating PGCs upon interaction with cellular domains expressing different levels of E-cadherin. **a** Schematic representation of the experiments presented in the figure. Cell clones were generated by injecting fluorescently labeled morpholinos (green) and RNAs into one out of 32 blastomeres. The germ cells expressed LifeAct fused to mCherry. Embryos containing the labeled transplant were imaged between 7 and 9 hpf using light-sheet microscopy. The right panels show an example of a polarized PGC that migrates from the non-manipulated area (black) and contacts a MO-treated clone (green). Scale bar, 10 μm; white arrow in the inset indicates the direction of migration. **b**–**d** Snapshots showing examples of the three types of behaviors observed upon contact between PGCs and MO-treated clones (*e-cadherin* MO in these cases)*:* no reaction, change of polarity and loss of polarity (See Supplementary Movies 7, 8 and 9). The separate panels connected with dashed lines on the right provide magnifications of the cells (in **c** and **d**, rotated views are presented, with the direction of rotation indicated by the black curved arrows). White stars label the somatic cells contacted by the PGCs; white arrows indicate the direction of migration; min = minutes; *t* = 0 min indicates the moment of contact; yellow arrows point at the contact point; scale bars, 5 μm. Cells are derived from the same data sets presented in **e**. Snapshots in **b** correspond to time points 1 min, 2 min, 3 min (contact), 4 min, 5 min and 11 min in the Supplementary Movie 7. Snapshots in **c** correspond to time points 0 min, 1 min, 2 min, 3 min (contact), 4 min and 8 min in the Supplementary Movie 8. Snapshots in Fig. 5d correspond to time points 1 min, 2 min, 3 min (contact), 4 min, 5 min and 11 min in the Supplementary Movie 9. **e** Upper schematics explaining the generation of the two scenarios analyzed in the graph below. Graph: the percentage of PGCs showing no reaction (black striped columns) or change and loss of cell polarity (blue striped columns) upon contact with control clones or clones injected with *e-cadherin* MO. Mean ± s.e.m.; *P* value: two-tailed Mann–Whitney *U-*test; *n* = number of contact events from 6 independent experiments. Source data are provided as a Source Data file.

The model we propose above suggests that the polarity and the migration direction of single cells can be influenced by the E-cadherin level in the surrounding cells and tissues. To examine this prediction, we generated, within wild-type embryos, clones of somatic cells depleted of E-cadherin and analyzed the behavior of PGCs upon contacting those clones. The procedure for generating such clones is illustrated in Fig. 5a. Specifically, we injected one out of 32 blastomeres with either *control* or *e-cadherin* MOs, both tagged with a green fluorophore (Supplementary Fig. 10a, b). As the germ cells expressed a LifeAct-mCherry protein fusion, we were able to characterize the immediate effect that interaction with the cell clones had on PGC polarity. We classified the cell responses into three categories: no reaction when the cell maintained the same polarity and direction of migration (Fig. 5b and Supplementary Movie 7), change of polarity when the cell exhibited a persistent enrichment of actin, albeit at a different position of the cell front, leading to a change in the migration trajectory (Fig. 5c and Supplementary Movie 8), and loss of polarity when the cell showed only transient enrichment of actin at different locations and stopped migrating (Fig. 5d and Supplementary Movie 9). While the majority of PGCs (73.11% ± 7.27%) showed no reaction upon contacting control clones, those that contacted the clones with downregulated E-cadherin were strikingly affected, with 76.99% ± 6.34% of the PGCs changing or losing their polarity (Fig. 5e). Increased frequency of loss or change of polarity behaviors was not observed for PGCs that encounter cell clones containing both *e-cadherin* MO and an *e-cadherin* RNA insensitive to the MO (Supplementary Fig. 10c). Thus, the changes in PGC responses upon contacting the experimental clones were related to the E-cadherin content.

Together, these results independently support our proposed role for E-cadherin in sustaining actin polarization and are consistent with the idea that differences in adhesion levels in the cellular environment can account for distribution patterns of migrating cells in tissues. Indeed, when the cell front of a wild-type migrating cell comes into contact with a cellular domain that contains reduced levels of E-cadherin (Fig. 5), the cell experiences an abrupt and asymmetrical change in the amount of engaged E-cadherin molecules at its leading edge. As a result, actin flow velocity might differ within various parts of the cell front (as demonstrated for a different model^44^), leading to loss of polarity or to a change in the direction of migration. It would be interesting to further investigate the mechanisms that control the precise cellular response to such interactions. For example, the response could depend on the orientation of the contact point at the leading edge with respect to the entire cell front area, it could be influenced by the size of the contact area or it could be affected by the polarization state of the cell prior to the contact.

## Discussion

The migration of a variety of cell types relies on the formation of actomyosin-dependent protrusions called blebs. The precise effect of cell–cell interactions between bleb-driven migratory cells and cells in their environment is currently not well defined. Here, we employed zebrafish primordial germ cell as a model to study the role of cell–cell adhesion in bleb-driven single-cell migration in vivo.

Our results first show that E-cadherin-mediated cell–cell adhesion is primarily required for the persistence of PGC directional migration rather than for motility per se. Importantly, the model we propose regarding E-cadherin function does not rule out the possibility that E-cadherin does contribute to the generation of friction that is important for translocation of the cells forward. Even in such a case, the migration speed of cells with reduced E-cadherin levels (rather than a complete loss of E-cadherin and of other molecules with a redundant function) would not be necessarily affected. Indeed, depletion of E-cadherin is coupled to an increase in the speed of actin retrograde flow (Fig. 2c, d, f), which could in turn positively contribute to forward movement of the cell and compensate for the reduced friction as described in other cases^45^. It would be interesting to conduct further experiments to quantitatively test this hypothesis.

The distribution of protrusions at the leading edge of migrating cells controls the precise direction of their movement, as we demonstrate here also for migrating PGCs. While much is known concerning the composition and the processes driving the expansion of protrusions (e.g., actin polymerization and fluid flow), the mechanisms that fine-tune their distribution around the cell circumference are less understood. Chemotactic cues can initiate intracellular signalling events that bias the production of projections in a certain direction, coupled with spatially regulated biochemical feedback networks that restrict protrusion spread and maintain polarity^46^. For example, in neutrophils and *Dictyostelium discoideum*, positive feedback cascades at the cell front restrict the formation of actin-driven protrusions to the leading edge, while enzymatic activities at the cell rear inhibit actin polymerization^46^. In addition to the biochemical activity networks that generate and maintain polarity, restricting protrusion formation to the cell front can be controlled by differences in membrane tension^47^. Specifically, by unfolding wrinkles in the membrane, actin polymerization can increase membrane tension, which can then rapidly propagate and prevent the formation of secondary fronts elsewhere by long-range inhibition of actin assembly and Rac activation^48^. A role for membrane tension in controlling the site of protrusion formation has also been described in blebbing cells, where high membrane tension coupled with negative membrane curvature favors the formation of the protrusion at the flanks of pseudopods or other blebs^12,49^. Focusing blebbing activity at the cell front can also be achieved by polar distribution of membrane-cortex linker proteins^11^ and of PIP2 in the membrane^10^. These molecules inhibit the separation of the membrane from the cortex and the inflation of blebs, as shown, for example, in melanoma cells; here, high levels of the membrane-cytoskeleton linker ezrin at the uropode inhibits blebbing at the rear of the cells^50^.

Our in vivo observations in zebrafish PGCs lead us to put forward a different molecular mechanism for focusing protrusive activity at the leading edge of single migrating cells. According to the model presented in Fig. 4a, this mechanism depends on E-cadherin-mediated cell–cell adhesion that limits actin enrichment at the cell front. Importantly, the effect of E-cadherin on the distribution of actin does not involve biochemical signalling, as we found that the activity level of the GTPase Rac1 was not affected by E-cadherin knockdown (Supplementary Fig. 5a, b). In polarized PGCs, the distribution of actin around the cell perimeter strongly correlates with blebbing activity (Fig. 3b). As actin and myosin are known to interact and are enriched at the leading edge of PGCs (Fig. 3a and Supplementary Fig. 7a), we propose that actomyosin-based contractility favors bleb formation within this region (named “Bleb-Prone Region” or “BPR”, green in Fig. 4a). Accordingly, when the function of E-cadherin is knocked down, actin distribution at the cell front is less focused, leading to an expansion of the BPR. An additional non-mutually exclusive mechanism by which actin distribution could influence the position of bleb formation would involve an effect on membrane tension and curvature, in accordance with findings in *Dictyostelium discoideum*^12,49^. Our findings highlight the importance of determining the distribution and activation pattern of different types of myosin molecules in migrating cells, thereby obtaining a comprehensive view of the cell’s contractility profile and its correlation with cell behavior.

We show that by altering the level or activity of E-cadherin and of E-cadherin-actin linkers cell migration paths can be regulated. This principle is especially pronounced when the migrating cells encounter domains of different adhesion levels. The intracellular events caused by the interaction of cells with such structures are translated into changes in their migration path that can thus account for the immediate response and the long-term distribution patterns of the cells in tissues. Interestingly, we have previously reported that the interaction of PGCs with the developing gut compromises cell polarity and leads to cell migration away from the tissue, ultimately controlling PGC positioning^51^. It would be interesting to investigate whether the adhesion properties of this specific tissue and of other types of cell migration barriers could indeed explain the behavior of cells that interact with them.

An additional intriguing issue concerns a phenotype we previously described for an overexpression of E-cadherin, which resulted in defects in cell polarization^52^. While we show here that E-cadherin supports polarized actin enrichment and focused blebbing activity, very strong elevation in E-cadherin levels could interfere with the dynamic distribution of actin and with the generation of one dominant actin-rich domain at the cell front. Thus, while alterations in cell–cell adhesion qualities can control the physical properties of whole tissues, they can also regulate features related to the ability of cells to polarize, for example in the context of migration. This issue is particularly relevant in processes where cells individualize as a result of changes in cell–cell adhesion. In such events, for example in cancer metastasis and epithelial-to-mesenchymal transition (EMT) processes, alterations in the expression of cell adhesion molecules allow cells to dissolve stable interactions with other cells. At the same time, if cells maintain the correct adhesion level, this can allow them to preserve the dynamic front-rear polarity characteristic of individually migrating cells.

## Methods

### Zebrafish strains

Zebrafish (*Danio rerio*) of the AB and AB/TL backgrounds and transgenic fish carrying the *kop:mcherry-f-nos3*′*UTR* (expressing farnesylated mCherry protein in the PGCs)^25^, *kop:lifeact-egfp-nos3*′*UTR* transgene (expressing LifeAct-EGFP in the PGCs)^27^ and the *kop:lifeact-mCherry-nos3*′*UTR* transgene (expressing LifeAct-mCherry in the PGCs, this work) were used as wild-type fish. To generate the transgenic line *kop:lifeact-mCherry-nos3*′*UTR*, a plasmid containing the *kop* promoter^53^, LifeAct-mCherry coding sequence, the *nanos3*′*UTR*, the dominant marker cry:DsRed, and the Tol2 sites^54^ was generated using Gateway Technology (Invitrogen). The purified plasmid was injected into one-cell zebrafish embryos and adult female fish carrying the transgene were identified by screening for the dominant marker. *weg*^*tx230*^ mutant embryos^17^ were used for investigating the function of E-cadherin in the experiments indicated.

Fertilized eggs were collected and raised at 25, 28 or 32 °C. Embryos were kept in 0.3× Danieau’s solution [17.4 mM NaCl, 0.21 mM KCl, 0.12 mM MgSO_4_·7H_2_O, 0.18 mM Ca(NO_3_)_2_, 1.5 mM HEPES (pH 7.6)]. The general fish maintenance at the Institute follows the regulations of the LANUV NRW and is supervised by the veterinarian office of the city of Münster. Zebrafish were maintained according to the regulations described in ref. ^55^.

### Plasmid cloning, mRNA synthesis and embryo microinjection

One-cell stage Zebrafish embryos were microinjected into the yolk with mRNAs and translation-blocking morpholino antisense oligonucleotides (MOs; Gene Tools, LLC). For the clone experiments, fluorescently labeled MOs (fused to carboxyfluorescein at the 3′ end) were injected into one blastomere of 32-cell stage embryos. Capped mRNA was synthesized using the mMessage Machine kit (Ambion, Germany) according to the manufacturer’s instructions. Expression of proteins in germ cells was achieved by cloning the corresponding open reading frames (ORFs) upstream to the 3′ untranslated region (UTR) of the *nanos1* gene (*nos3*′*UTR)*^56^, whereas for ubiquitous expression, the *Xenopus globin3*′*UTR* was used. For rescue experiments of the *e-cadherin* MO and *e-cadherin* fluorescent MO, an *e-cadherin* RNA that contains 6-base pairs sequence mismatch compared with the morpholino sequence and that includes the *Xenopus globin3*′*UTR* was used. This RNA was co-injected with the morpholino antisense oligonucleotides at the molarity indicated in Supplementary Fig. 1e and in Supplementary File 1B, D. For details about the experimental designs, the primers used for DNA cloning, the mRNAs and MOs used in this work, and the injected amounts see Supplementary File 1A–D. Control and experimental mRNAs and experimental MOs were always injected at equimolar amounts (if needed, control mRNA and control MO were added to the injection solution to reach equimolarity). As the RNA molecules differed in size, the actual picogram amounts injected were not identical in the control and experimental embryos.

### Imaging

Prior to image acquisition, embryos were hand dechorionated and those 22 hpf old were anesthetized using 0.64 mM Tricaine (Sigma) in 0.3× Danieau’s solution. Embryos were transferred to agarose ramps covered with Danieau’s solution and oriented, except for the experiments with morpholino-treated clones where embryos were ramped in 1% low melting point agarose in translucent tubes. During live imaging, sample temperature was kept constant using a heated stage (PECON, TempController 2000–2).

#### Migration track analysis

Time-lapse imaging was performed using an Axio.Imager.M1 (Zeiss) and an Axio.Imager.Z1 (Zeiss) controlled by the VisiView® software (versions 2.1.4 and 4.2.0.2, Visitron Systems, GmbH). Imaging was performed using a ×10 water-immersion objective (Zeiss, NA 0.3) at a rate of one frame per 2 min over 120 min in total, starting at 6 hpf. Three focal planes (50 μm apart) were imaged to generate Z-stacks.

#### Assessment of somatic phenotypes

Imaging was performed on 10 hpf embryos using a LeicaMZ16F microscope equipped with an AxioCam MRc5 (Zeiss) and controlled by the ZEN software (version lite 2012, Zeiss, Germany).

#### Subcellular localization of DN E-cadherin-EGFP

Imaging was performed on 8 hpf embryos using an LSM710 confocal microscope (×20 and ×40 water-immersion objectives, Zeiss, NA 0.5 and NA 0.75, pinhole 69 µm, 1024 × 1024) controlled by the ZEN software (version 2010 B SP1, Zeiss, Germany).

#### Subcellular localization of E-cadherin-EGFP in the PGCs

Time-lapse imaging was performed on 7–9 hpf embryos using a Zeiss AxioImager.M2 microscope controlled by the VisiView® software (version 4.0.0.14, Visitron Systems, GmbH) and equipped with a dual view filter (MAG Biosystems, Exton, PA), Photometrics cameras (Cascade II and CoolSNAP ES2) and VS-Laser Control. Frames were captured at 2 s intervals using a ×63 water-immersion objective (Zeiss) with 488 and 561 nm lasers and 500 and 200 ms exposure times, respectively.

#### Whole-mount immunohistochemistry

Imaging was performed on 10 hpf fixed embryos using a Zeiss AxioImager.M2 microscope controlled by the VisiView® software (version 4.0.0.14, Visitron Systems, GmbH) and equipped with a dual view filter (MAG Biosystems, Exton, PA), Photometrics cameras (Cascade II and CoolSNAP ES2) and VS-Laser Control. Frames were captured using a water-immersion ×40 objective (Zeiss, NA 0.8) with a step size of 2 µm.

#### Actin signal intensity profile

Live imaging was performed on 7–9 hpf embryos using an LSM710 confocal microscope (×40 water-immersion objective, Zeiss, NA 0.75, pinhole 154 µm, 512 × 512, 15 s per frame) controlled by the ZEN software (version 2010 B SPI, Zeiss, Germany).

#### Dynamics and distribution of actin and blebs within the cell

Time-lapse imaging was performed on 7–9 hpf embryos using a Zeiss AxioImager.M2 microscope controlled by the VisiView® software (version 4.0.0.14, Visitron Systems, GmbH) and equipped with a dual view filter (MAG Biosystems, Exton, PA), Photometrics cameras (Cascade II and CoolSNAP ES2) and VS-Laser Control. Frames were captured at intervals of 500 ms for E-cadherin knockdown experiments and 1 s for β-catenin inhibition experiments using a ×63 water-immersion objective (Zeiss) with 488 and 561 nm lasers and 200 ms exposure time.

#### Rac1- and RhoA-FRET

Live imaging was performed on 7–9 hpf embryos using an LSM710 confocal microscope (×40 water-immersion objective, Zeiss, NA 0.75, pinhole 280 µm, 512 × 512, 7.75 s per frame) controlled by the ZEN software (version 2010 B SP1, Zeiss, Germany).

#### Subcellular localization of myosin light chain 12.1-EGFP

Live imaging was performed on 10–14 hpf embryos using a Zeiss AxioImager.M2 microscope controlled by the VisiView® software (version 4.0.0.14, Visitron Systems, GmbH) and equipped with a dual view filter (MAG Biosystems, Exton, PA), Photometrics cameras (Cascade II and CoolSNAP ES2) and VS-Laser Control. Frames were captured at 2 s intervals using a ×40 water-immersion objective (Zeiss) with 488 and 561 nm lasers and 600 and 300 ms exposure times, respectively.

#### Actin and blebs distribution in PGCs expressing Cxcl12a

Time-lapse imaging was performed on 7–9 hpf embryos using an Axio.Imager.Z1 (Zeiss) controlled by the VisiView® software (version 4.2.0.2, Visitron Systems, GmbH) and equipped with Photometrics camera Prime 95B and VS-Laser Control. Frames were captured at 5 s intervals using a ×40 water-immersion objective (Zeiss, NA 0.75) with 488 and 561 nm lasers and 300 and 400 ms exposure times, respectively.

#### MO-treated clones experiments

Live imaging was performed on 7–9 hpf embryos using a Zeiss Lightsheet Z.1 microscope equipped with a ×20 water-immersion objective, 488 and 561 nm lasers, and controlled by the ZEN software (version 2014 SPI, Zeiss, Germany). Up to eight embryos were ramped in 1% low melting point agarose in translucent tubes (Fluidflon FEP-Schlauchabschnitt 1.6 × 2.4 mm (Wd: 0.4 mm)) which were covered with methylcellulose. The chamber of the microscope was filled with 0.3× Danieau’s buffer and heated to 28 °C. Time-lapses were acquired with a time resolution of 1 frame per minute and a z-resolution of 2 µm. For the acquisition, single side illumination was employed and a zoom of 0.8% was used.

### Migration track analysis

Images were acquired and Z-projected using the MetaMorph (Molecular Devices, LLC, version 7.7.9.0 2012) package. The ‘spots’ module of the Imaris software (Bitplane, Switzerland) was used for tracking of PGCs and for correction for gastrulation-derived tissue movement. In this procedure, we made use of the labeled nuclei of somatic cells located closest to the tracked germ cells, thereby taking into consideration the effect of whole tissue movement on PGC tracking and speed measurements.

### Counting of ectopic PGCs

To calculate the number of ectopic germ cells, PGCs expressing LifeAct-EGFP were counted at 22 hpf in the GFP-channel using a ×20 water-immersion objective.

### Whole-mount immunohistochemistry

Detection of E-cadherin in zebrafish embryos was performed as previously described^53^, using a polyclonal mouse antibody directed against the protein (610181, BD Biosciences) at a 1:200 dilution and detected using a fluorophore-conjugated secondary antibody (Alexa Fluor® 546F(ab′)_2_ fragment of goat anti-mouse IgG, Invitrogen). The nuclei of all cells were labeled by incubating the embryos for 30 min in a 1:10,000 Hoechst dilution in PBT in the dark with gentle agitation. Images were processed using Fiji/ImageJ software. Z-stacks of the embryos were acquired and the first 10 images were subsequently merged into an average intensity projection using an automated Fiji plugin. The mean intensity of an area of a defined size in the centre of the Z-projection was measured. As control for unspecific binding of the fluorophore-conjugated secondary antibody, embryos from the same egg clutches were incubated in blocking solution without the primary antibody. The average fluorescence value of this control was subtracted from the values of all other samples. For each condition, the values of the E-cadherin depleted samples were normalized to the respective controls.

### Subcellular localization of E-cadherin-EGFP in the PGCs

To analyse E-cadherin distribution around the cell perimeter in PGCs, cells expressing both E-cadherin-EGFP and farnesylated mCherry proteins were used. To correct for differences in E-cadherin signal level that stem from variabilities in membrane density and shape, E-cadherin distribution was considered to be the ratio between E-cadherin and the membrane signals. For this analysis, only motile cells (showing a net displacement) were imaged. Single frames in which the cell front and rear appeared simultaneously in focus were subjected to analysis using ImageJ (2.0.0-rc-69/1.52p, NIH) as described below.

First, the outlines of both E-cadherin and membrane signals were derived as follows. For each channel, images were processed with the Enhance contrast function in the Process section to saturate 0.1% of pixels. An outline of the signal was manually generated using the Segmented line tool (line width = 1 pixel). The line was drawn in between the two brightest pixels and its starting point was set at the beginning of the leading edge. Increments were added counter clockwise until the starting point was reached again.

The intensity distribution profiles for E-cadherin and membrane signals were then derived by applying to the original unprocessed images the outlines obtained as described above. Intensity distribution was displayed via the Plot Profile function in the Analyse section. List was selected to derive intensity values.

Last, the length of each cell outline was normalized to 1 and the ratio between the intensity values obtained for E-cadherin and membrane signal was calculated and then normalized to the mean intensity value.

### Actin signal intensity measurements

Germ cells expressing LifeAct-EGFP and farnesylated mCherry proteins were used. Polarized (showing an actin enrichment at one aspect of the cell) and motile (showing a net displacement) cells were imaged. Only frames in which the cell front and rear appeared simultaneously in focus (based on the membrane signal, farnesylated mCherry) were further analyzed.

To generate actin signal intensity profiles, an ImageJ (V.1.51, NIH) plugin was designed. The input for the plugin is a 16-bit grayscale image of the cell of interest, that has been background corrected and thresholded using Fiji software. In this way, irrelevant pixels were designated as possessing intensity values of 0. When required, images of cells were rotated to ensure proper orientation along the *y*-axis, with the cell front facing up. The plugin reads and averages the intensities of all relevant pixels in a line along the *x*-axis. The average values of each line were exported as a table for subsequent analysis and presented as intensity profiles along the *y*-axis of the original images. To generate the mean curves, the cell length was normalized into 50 segments and the signal intensity level was normalized to the cell area.

To calculate the ratios between the mean value of LifeAct-EGFP signal intensity at the cell front (F) and in the middle part of the cell (M), the same 16-bit input images used for the signal intensity profiles were utilized but thresholded using Fiji software in a way that no value was attributed to irrelevant pixels. The cells were then divided into five sectors according to their length. The ratio between the mean value in segment 1 (F) and that in segment 3 (M) was then calculated for each cell.

### Rac1- and RhoA-FRET analysis

Analysis was performed using ImageJ and Fiji software as previously described^25^, with the difference that the FRET ratios for the whole-cell measurements were obtained for 15 frames for each cell.

### Kymographs

Actively migrating cells expressing LifeAct-EGFP and farnesylated mCherry proteins were analyzed. Movies were captured at the focal plane of the cell membrane signal, thereby ensuring that both the front and the rear were in focus. Analysis was performed using Fiji. For each movie, 50 time points at intervals of 500 ms were analyzed. First, all cells were rotated to ensure their orientation along the *y*-axis (cell front to the left). A line (width 4) was drawn along the axis of cell polarization and kymographs were obtained using the Dynamic Reslice command. 1-dimensional (1D) retrograde cortical flow speeds were extracted by manually measuring the angle α generated between every visible bright line in each kymograph and the cell front-rear axis. To analyse the movement of actin only at the cell cortex and its proximity, time points of blebbing events were excluded from the analysis and measurements were performed within an area located at a maximum distance of 2 μm from the cell border into the cell. When needed, to highlight single lines in the kymographs the Find edges command was employed. The derived angles *α* were finally used to calculate velocities as *v* = [(μm/pixel) * (seconds/frame)] * [1/tang(*α*)].

### Quantification of actin flow

#### Cell segmentation and intracellular motion fields (BioFlow)

Analysed cells were derived from the same sets of cells used in the kymograph analysis. Individual cells were segmented using an active contour model that accounts for region intensity and edge information. The modified optical flow technique described in ref. ^35^ was then employed to estimate velocity fields ***v***(***x***, *t*) within the segmented cells, ${\boldsymbol{x}} \in {\cal{C}}\left( t \right)$, from successive fluorescence images. The method, automated in the software BioFlow^35^ within the Icy^57^ platform, is especially robust concerning out-of-plane motion and missing information.

When the flow-field analysis was performed for two channels at a time (cytoplasm and actin or E-cadherin and actin), since the signal pairs were uncoupled, we considered each channel separately and obtained independent velocity measurements for the movement of each protein. In the case of filamentous actin, only one channel was used.

#### Tracking and integration of actin structures

While the cell and its inner velocity field are available, following the actin enrichment at the cell front ${\cal{B}}\left( t \right) \subset {\cal{C}}\left( t \right)$ over time is challenging because the structure is very diffused, precluding the use of a standard segment-&-track approach. Therefore, we automatically set an initial sub-region ${\cal{B}}_0$ on the leading edge of the cell, where the structure is located at an initial time *t*_0_. ${\cal{B}}_0$ can be tuned by width and arc length to better fit the structure of interest. To compute ${\cal{B}}\left( t \right)$, we advect ${\cal{B}}_0$ through time by solving the ordinary differential equation (ODE) posed by the velocity field:

1 $$\dot {\cal{B}} = {\boldsymbol{v}}({\cal{B}},t),\quad {\cal{B}}(t = t_0) = {\cal{B}}_0.$$

To assess the speed and displacement of the moving actin in the direction of the cell polarization axis, we tailor a measure that captures this motion quantitatively. We propose to use the average velocity $\bar v$ of the actin enrichment ${\cal{B}}\left( t \right)$ projected on the axis ***p*** of cell polarity. The average is taken over a time lapse ∆*T* before the bleb occurs at *t* = *T*_bleb_:

2 $$\bar v \ := \frac{{\bar d}}{{{\Delta} T}},$$

3 $${\bar {d}} \ := \mathop{{{\smallint }}}\limits_{T_{{\mathrm{bleb}}} - {\Delta} T}^{T_{{\mathrm{bleb}}}} \left| {{\cal{B}}\left( t \right)} \right|^{ - 1}\mathop {\iint}\limits_{{\cal{B}}\left( t \right)} {{\boldsymbol{v}}\left( {{\boldsymbol{x}},t} \right) \cdot {\boldsymbol{p}}{\mathrm{d}}{\cal{B}}{\mathrm{d}}t} ,$$

where *t*_0_ is set to $T_{{\mathrm{bleb}}} - {\Delta} T$ in the ODE. By definition, $\bar v$ is proportional to the total backwards displacement $\bar d$ that actin undergoes (during ∆*T*) with respect to the front-to-rear polarization axis.

The methodology can be applied to any other structure such as the actin cortex ${\cal{A}}\left( t \right) \subset {\cal{C}}\left( t \right)$. To analyse spatial differences within this region, we further sub-divide it into *n* evenly spread (according to arc length) partitions ${\cal{A}}_i\left( t \right) \subset {\cal{A}}\left( t \right),i \in \{ 1..n\}$; for example, to compare the average projected velocity at the cell front with that at the sides or at the rear. In this case, the measure remains practically unchanged:$\bar v_i\ := \bar d\left( {{\cal{A}}_i} \right)/{\Delta} T$, but the borders between regions have to be treated with care during advection.

To study actin flow around the whole cell, we divided the cortex into *n* = 4 equally-spaced regions corresponding to the front, the centre-front, the centre-back, and the back of the cell (regions 1, 2, 3 and 4, respectively, in Fig. 2f and Supplementary Fig. 5f). In Supplementary Fig. 5d, e only the front part was used. The comparison between the movement of F-actin and that of E-cadherin was also done at the cell front (Supplementary Fig. 5c).

In all cases, the regions were defined to have a thickness of 1.7 μm from the cell boundary inwards. The flow of actin was integrated over ∆*T* = 10 s preceding each bleb to yield an averaged velocity. Sliding the time interval back and forth by a moderate amount did not change the measured values significantly, nor did adjusting the thickness. This perturbation analysis shows that our method is robust. To enhance the objectivity of the measurements, the axis of cell polarization ***p*** was automatically adjusted using an intensity-weighted version of principal component analysis. In this way, the axis corrected itself with the appearance of new blebs. Setting the axis manually for each individual bleb yielded very similar measured values.

The tool presented in this section is available as a custom-made Python script that uses four main libraries: SciPy^58^ and NumPy^59^ to solve the advection problem and implement a composite trapezoidal time integral, and FEniCS^60^ and CGAL^61^ to handle the regions as triangulated meshes, and cast the finite elements used for the spatial integral.

### Subcellular localization of myosin light chain 12.1 in the PGCs

Polarized (showing actin enrichment at one aspect of the cell) and motile (showing a net displacement) cells co-expressing Myl12.1-EGFP and LifeAct-mCherry were imaged. Movies were captured at the focal plane of the actin signal. Time-lapse images of the cells were acquired on 10–14 hpf embryos (older than those used in most of the experiments to allow the signal of the Myl12.1-EGFP to become stronger).

### Correlation between actin and bleb distribution within PGCs

Actively migrating cells expressing LifeAct-EGFP and farnesylated mCherry proteins were analyzed. Movies were captured at the focal plane of the cell membrane signal, thereby ensuring that both the front and the rear were in focus. For each movie, 490 time points at intervals of 500 ms were analyzed.

Simulations were performed with a home-written Python code.

In short, the required input was a fluorescence image consisting of a membrane channel and an actin channel. Both images were blurred with a Gaussian filter with *σ* = 3 pixels, to remove noise on a pixel level. The membrane image was then thresholded using an Otsu-filter, and the contours of the cell membrane were determined from the binary image. This was then used as a mask for the actin signal, as well as to determine the centre of mass of the cell.

From the actin fluorescence image both the entire cell area and the perimeter of the cell were angularly integrated. The front of the cell (defined as 0°) was determined as the angle of the maximum actin signal. This angle was smoothened over time with a *σ* = 25 s Gaussian filter to follow changes of direction of the cell, but to be insensitive to slight frame-to-frame variations. Blebs were identified as local maxima in the velocity of the centre of mass, whereas their angle with respect to the front was determined by the maximum edge velocity at that time point.

### Actin and blebs distribution in apolar PGCs

PGCs were engineered to express the guidance cue Cxcl12a by injection of *cxcl12a-nanos3*′*UTR* mRNA into transgenic zebrafish embryos whose PGCs expressed markers labelling actin (LifeAct-EGFP) and membrane (farnesylated mCherry) structures. This treatment generated a high level, uniform distribution of the chemokine that induces an apolar state of the cells. The RNA encoding for a red nuclear marker (*mCherry-nls*-*nos3*′*UTR)* was co-injected with *cxcl12a* mRNA to identify PGCs that produce the chemokine.

### Measurements of protrusions frequency and coordination

Bleb frequency was determined by manually counting protrusion events in polarized, migrating cells over 250 frames acquired at a time interval of 500 ms for E-cadherin knockdown experiments and 1 s for β-catenin inhibition experiments. The polarized state of the cells was confirmed by the co-expression of LifeAct-EGFP in the PGCs.

To calculate the angle between consecutive blebs, Fiji software was used. The multi-line ROI tool was used to manually select points along the fluorescently labeled cell membrane at the moment of initiation of a bleb. The centre of mass of the selected ROI was then determined and connected to the middle point of the bleb neck. The same procedure was repeated for the subsequent bleb and the angle between the two lines was determined.

### Friction-based transport model describing actin distribution

To describe the spatial distribution of the actin fluorescence signal along the front-rear axis of the cell we use a transport-decay model, where the F-actin network is transported retrogradely with velocity *v* while polymerizing with a constant rate *k*_+_ and depolymerizing with a constant rate *k*_−_. Here we only describe the region of retrograde transport, where a reduced polymerization is expected, and the depolymerization dominates. Directly at the leading edge, larger polymerization rates are taking place.

Conservation of mass dictates that the gradient of material flux $j = v \times c_a$ equals the temporal change in F-actin concentration $c_a$:

4 $$\frac{{dj}}{{dx}} = \frac{{dc_a}}{{dt}}$$

where *x* denotes the position in the cell along the front-rear axis. While moving towards the back of the cell, the network is depolymerized. Assuming a constant depolymerization leads to the rate equation:

5 $$\frac{{dc_a}}{{dt}} = - k_ - \times c_a(t)$$

Combining Eqs. (4) and (5) while assuming a constant flow velocity results in a first-order differential equation for the spatial distribution of the actin: $v\frac{{dc_a\left( x \right)}}{{dx}} = - k_\_ \times c_a\left( x \right)$, with the solution $c\left( x \right) = c_0\exp \left( { - \frac{x}{\lambda }} \right)$, where *c*_0_ is the peak actin concentration and $\lambda = v/k_ -$ denotes the decay length of the F-actin network, and $\frac{{k_ - }}{{k_ + }}$ describes the equilibrium concentration towards which the system develops.

Using a Levenberg–Marquardt algorithm in Matlab (Mathworks, USA), *λ* was determined by fitting the exponential decay to the experimentally measured fluorescence intensity of the LifeAct signal (Supplementary Fig. 4a, d). The fitting regime started 2 µm from the peak of the actin intensity until the actin signal decreased below 50% of the peak intensity (Fig. 4c and Supplementary Fig. 9).

As the velocity $v = \frac{{F_{{\mathrm{contraction}}}}}{\gamma }$ depends on the myosin mediated contraction and the E-cadherin mediated friction $\gamma \propto c_{{\mathrm{ecad}}}$ this model predicts $\lambda = \frac{{F_{{\mathrm{contraction}}}}}{{\gamma \times k_ - }}$ and hence an inverse relation between the decay length and the friction $\lambda \propto \gamma ^{ - 1}$.

### Morpholino-treated clone experiments

Cell clones were generated by injecting fluorescently labeled morpholinos and RNAs into one out of 32 blastomeres of embryos derived from *kop:lifeact-mCherry-nos3*′*UTR* transgenic females. Embryos containing the labeled transplant were imaged using light-sheet microscopy. Time-lapse movies were acquired at a time resolution of 1 min and a *z*-resolution of 2 µm. *x* and *y*-resolution were 0.286 µm. In the processing step, the *x*- and *y*-resolution was lowered to 0.572 µm. To improve the definition of the moment of contact between PGCs and the morpholino-treated clones, the surfaces of the MO-treated clone and of all germ cells were rendered using the surface plugin of the Imaris software (Bitplane). To determine whether PGCs were in a polar or apolar state, the Lifeact-mCherry signal was used. The behavior of germ cells upon contact with a MO-treated clone was defined as no reaction, change of polarity or loss of polarity based on the reaction of the PGC in the subsequent 10 frames. The Imaris software was also used to generate Movies 7, 8 and 9 and the snapshots presented in Fig. 5b–d. To this end, the animation option was used and an orthogonal view was employed.

### Statistical analysis

Figures where statistics was used represent pooled data from independent biological replicates as indicated in the figure legends. Appropriate controls were included for each biological replicate. Analyses were performed using GraphPad Prism and MATLAB. Normality of data was tested using the D’Agostino-Pearson omnibus normality test. Two-sided Student’s *t-*test was used for normal distributions and two-tailed Mann–Whitney *U-*test or two-tailed Kolmogorov–Smirnov test was applied in case of non-normal distributions with significance levels of *P < 0.05, **P < 0.01, ***P < 0.001 and ****P < 0.0001. No statistical method was used to predetermine the sample size. The experiments were not randomized and investigators were not blinded to allocation during experiments and outcome assessment.

### Reporting summary

Further information on research design is available in the Nature Research Reporting Summary linked to this article.

## Supplementary information

Supplementary Information

Description of Additional Supplementary Files

Supplementary Movie 1

Supplementary Movie 2

Supplementary Movie 3

Supplementary Movie 4

Supplementary Movie 5

Supplementary Movie 6

Supplementary Movie 7

Supplementary Movie 8

Supplementary Movie 9

Reporting Summary

## Data Availability

The data that support the findings of this study are available from the corresponding author upon request.

## Source data

Source Data

## Code availability

The source code used for the generation of the actin signal intensity profiles, for the analysis of actin flows using the BioFlow software, and for the study of the correlation between actin and blebs distribution around the cell perimeter will be made available upon request.
