# Supplementary material for: E-cadherin focuses protrusion formation at the front of migrating cells by impeding actin flow
Source: Nat Commun. 2020 Oct 26;11:5397. doi: 10.1038/s41467-020-19114-z (PMC7588466; doi:10.1038/s41467-020-19114-z)
Supplement: Supplementary file 1 — Supplementary Information [file 41467_2020_19114_MOESM1_ESM.pdf]

Supplementary Information

E-cadherin focuses protrusion formation at the front of migrating cells by impeding actin flow

Cecilia Grimaldi[1], Isabel Schumacher[1,+], Aleix Boquet-Pujadas[2,3,4 +], Katsiaryna Tarbashevich[1], Bart Eduard Vos[1], Jan Bandemer[1], Jan Schick[1], Anne Aalto[1], Jean-Christophe Olivo-Marin[2,3], Timo Betz[1] and Erez Raz[1*]

[+] These authors contributed equally
[*] Correspondence: erez.raz@uni-muenster.de

This file includes:

- Supplementary Fig. 1
- Supplementary Fig. 2
- Supplementary Fig. 3
- Supplementary Fig. 4
- Supplementary Fig. 5
- Supplementary Fig. 6
- Supplementary Fig. 7
- Supplementary Fig. 8
- Supplementary Fig. 9
- Supplementary Fig. 10
- Supplementary File 1A
- Supplementary File 1B
- Supplementary File 1C
- Supplementary File 1D
- Supplementary References

Legends for Supplementary Movies 1, 2, 3, 4, 5, 6, 7, 8, and 9 are provided in a separate PDF file entitled “Supplementary Movies legends”.

Supplementary Fig. 1

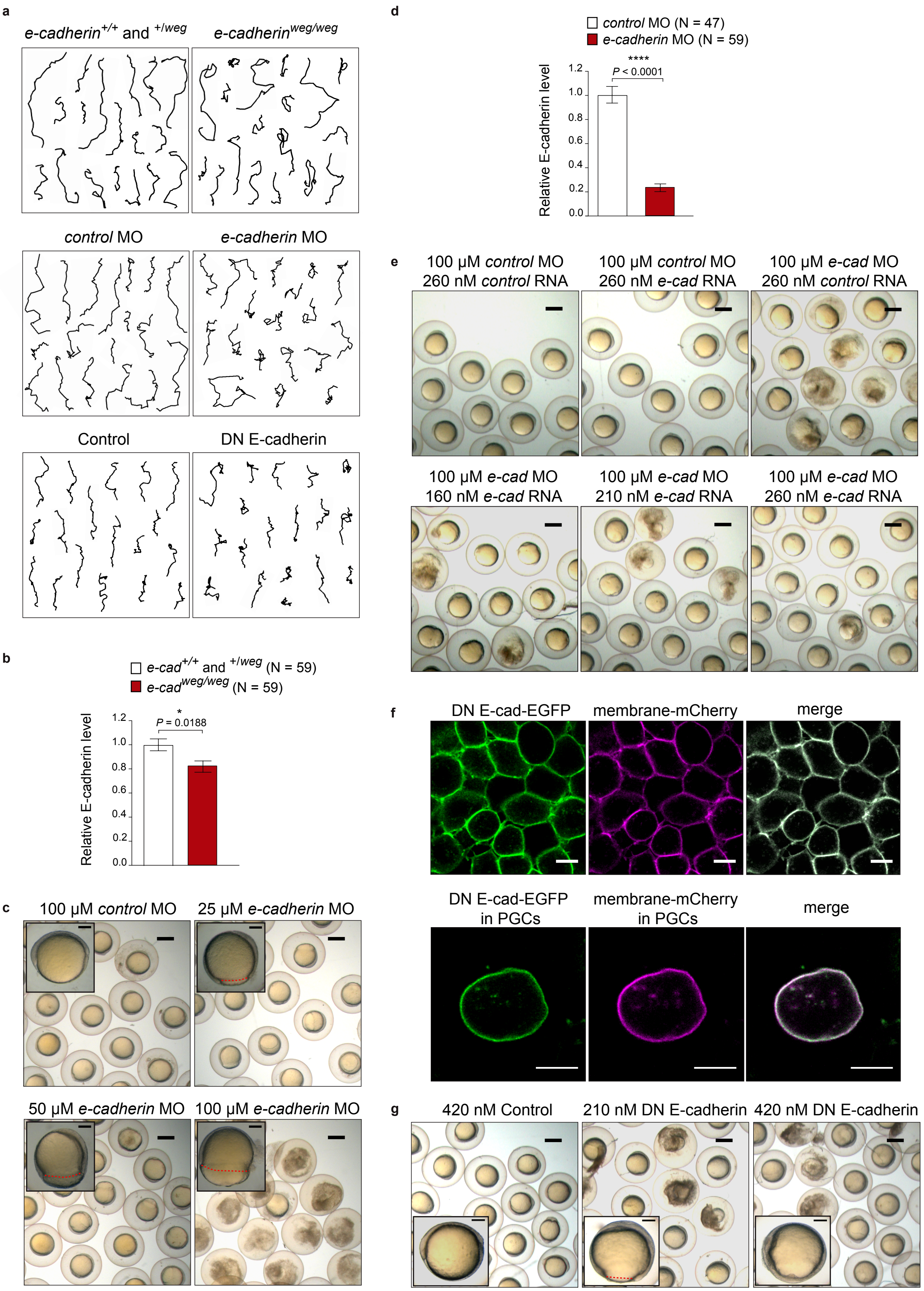

Supplementary Fig. 1: Characterization of the tools utilized for E-cadherin depletion. a, Representative tracks of PGCs migrating within *e-cadherin*$^{+/+}$ and $^{+/weg}$ embryos or *e-cadherin*$^{weg/weg}$ embryos (upper panels), migrating within embryos injected with a *control* or *e-cadherin* morpholino (middle panels), and overexpressing a control molecule or a dominant negative version of E-cadherin (lower panels). Data are derived from the same sets of cells analysed in Fig. 1a, b, c. **b,** Changes in E-cadherin levels in *e-cadherin*$^{weg/weg}$ embryos (red column) relative to their siblings *e-cadherin*$^{+/+}$ and $^{+/weg}$ (white column). Embryos were fixed at 10 hours post fertilization (hpf) and stained for E-cadherin. N = number of embryos pooled from 4 independent experiments. Normalized mean ± s.e.m.; *P* value: two-sided Student's *t*-test. **c,** Titration of morpholino-mediated knockdown of *e-cadherin*. Representative images of 10 hpf embryos injected either with *control* morpholino or with increasing amounts of *e-cadherin* morpholino. Scale bars, 500 μm. Inserts: red dotted lines mark gastrulation progression; scale bars, 150 μm. The experiment was repeated three times. **d,** Relative changes in E-cadherin levels in embryos injected with 100 μM of either *control* or *e-cadherin* morpholino. Embryos were fixed at 10 hpf and stained for E-cadherin. N = number of embryos pooled from 4 independent experiments. Normalized mean ± s.e.m.; *P* value: two-tailed Mann-Whitney *U*-test. **e,** Rescue of the phenotype induced by *e-cadherin* MO. Representative images of embryos co-injected with *control* MO and *control* RNA (upper, left panel), with *control* MO and *e-cadherin* RNA (upper, middle panel), with *e-cadherin* MO and *control* RNA (upper, right panel), or with *e-cadherin* MO and increasing concentrations of an *e-cadherin* RNA insensitive to the MO (lower panels). Embryos were imaged at 10 hpf. Scale bars, 500 μm. The experiment was repeated three times. **f,** Fluorescent images showing the subcellular localization of a dominant negative version of E-cadherin-EGFP expressed in all cells (upper left panel) or preferentially in PGCs (lower left panel). Scale bars, 10 μm. The experiment was repeated three times. **g,** Representative images of control embryos (left panel) and embryos injected with increasing concentrations of RNA encoding for a DN E-cadherin mutant that is translated in all cells (middle and right panel). Embryos were imaged at 10 hpf. Lower magnification images: scale bars, 500 μm. Inserts: red dotted line underlays gastrulation progression; scale bars, 150 μm. The experiment was repeated three times. Source data are provided as a Source Data file.

Supplementary Fig. 2

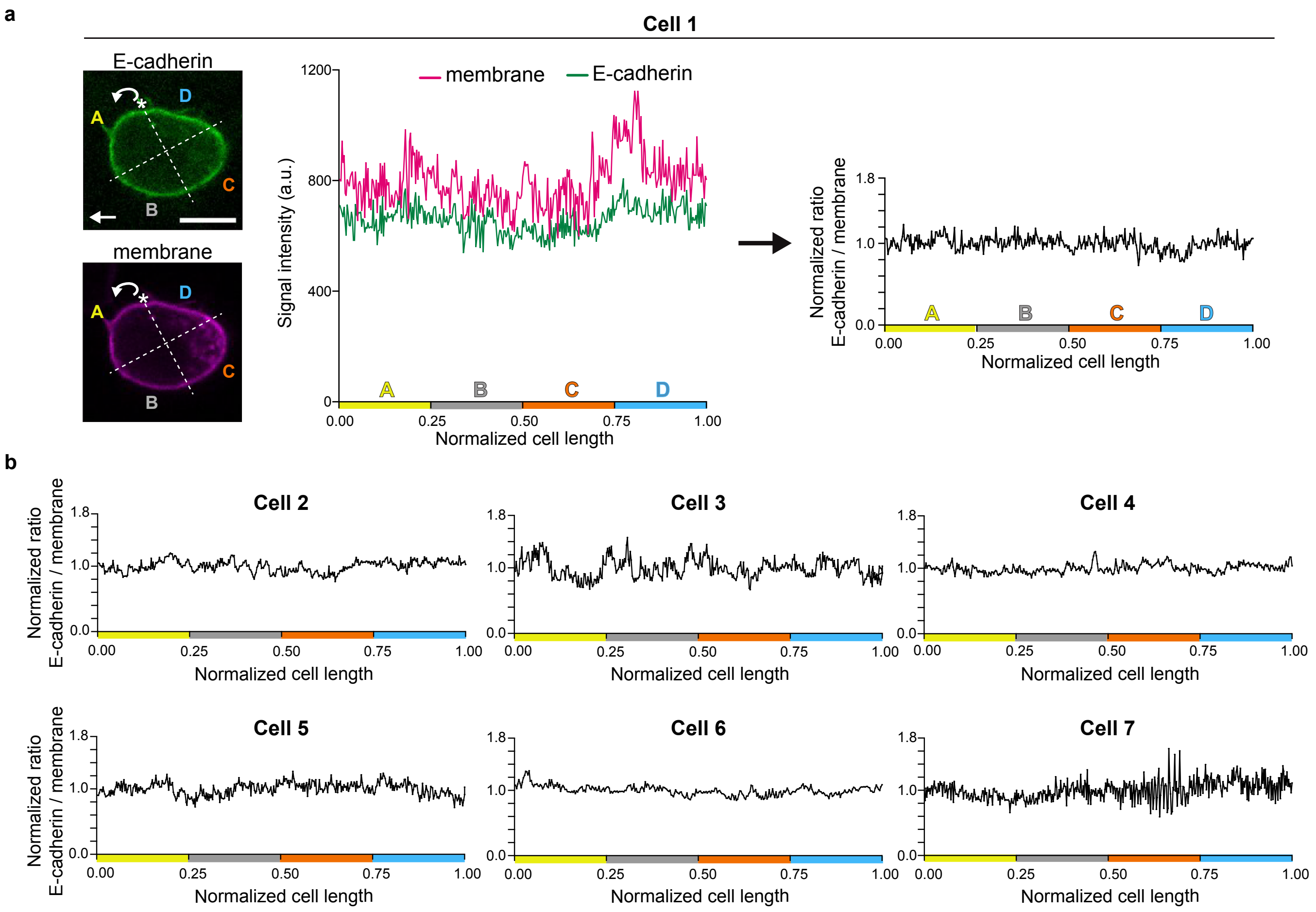

Supplementary Fig. 2: E-cadherin distribution around the cell perimeter of polarized PGCs. a, Snapshots on the left: representative cell expressing both E-cadherin-EGFP (green) and mCherry that contains a farnesylation signal, thereby labelling the cell membrane (magenta). The white stars indicate the starting point from which the outlines of E-cadherin and membrane signals were derived as described in the Methods section. Curved white arrows show the direction (counter clockwise) in which the outlines were drawn from the starting point. Dotted lines and coloured capital letters (A, B, C, and D) indicate the four segments into which the cell perimeter was divided. Straight white arrow indicates the direction of migration; scale bar = 10 μm. Left graph: signal intensity distribution profiles of E-cadherin (green) and membrane (magenta) around the cell perimeter. The four color-coded letters and segments correspond to the four regions of the normalized cell length indicated in the snapshots on the left. a.u. = arbitrary units. Right graph: visualization of E-cadherin distribution as the ratio between E-cadherin and the membrane signal normalized to the mean intensity. This presentation approach allows to correct for unspecific fluctuations in E-cadherin signal due to differences in membrane structure (e.g. folds). **b,** Graphs showing the normalized ratio between E-cadherin signal and that of the farnesylated mCherry protein in six additional PGCs. Source data are provided as a Source Data file.

Supplementary Fig. 3

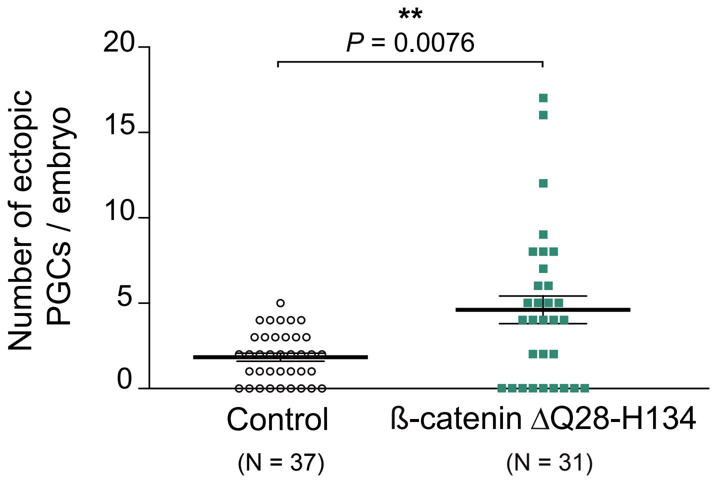

Supplementary Fig. 3: Effect of β-catenin inhibition on arrival of PGCs at the region where the gonad develops. Number of PGCs that reside out of the developing gonad region at 22 hpf in control PGCs and in cells expressing the mutated β-catenin protein. Mean ± s.e.m.; *P* value: two-tailed Mann-Whitney *U*-test; N = number of embryos from 3 independent experiments. Source data are provided as a Source Data file.

Supplementary Fig. 4

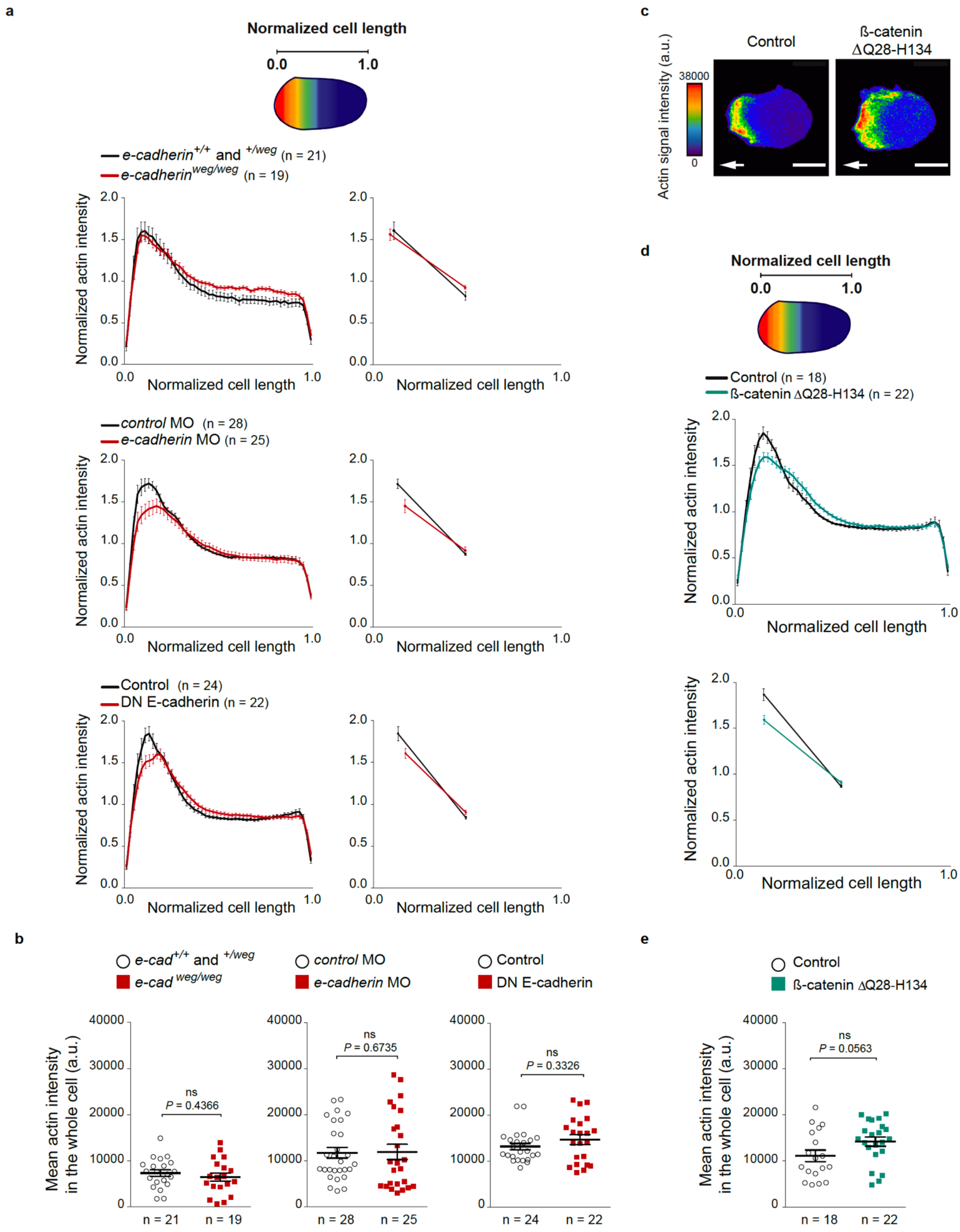

Supplementary Fig. 4: Actin distribution in PGCs upon E-cadherin and β-catenin knockdown. a, Upper panel: schematics explaining the measurements illustrated below. 0.0 = cell front and 1.0 = cell rear. Left graphs: mean normalized LifeAct-EGFP fluorescence intensity along the cell front-rear axis in PGCs located within control embryos and within embryos homozygous for a mutation in the *e-cadherin* locus (*e-cadherin*$^{weg/weg}$ mutants (upper graph)), in PGCs within *e-cadherin* morpholino treated embryos (middle graph), and in PGCs expressing the DN version of E-cadherin (lower graph)). Right graphs: lines connecting the highest and middle point of the corresponding graphs shown on the left. n = number of averaged curves analysed in 5 independent experiments for *e-cadherin*$^{weg/weg}$ mutants and 4 independent experiments for the other two conditions; mean ± s.e.m.. Data are derived from the same sets of cells analysed in Fig. 2b and Supplementary Fig. 4b. **b,** Mean values of LifeAct-EGFP intensity within the whole cell area. Open circles represent wildtype or control cells and red squares represent the intensity in PGCs within *e-cadherin*$^{weg/weg}$ mutant embryos (left graph), in PGCs within embryos treated with *e-cadherin* morpholino (middle graph), and in PGCs expressing the DN E-cadherin version (right graph)). n = number of cells from 5 independent experiments for *e-cadherin*$^{weg/weg}$ mutants and 4 independent experiments for the other two conditions; mean ± s.e.m.; *P* value: two-sided Student's *t*-test. Data are derived from the same sets of cells analysed in Fig. 2b and Supplementary Fig. 4a. **c,** Snapshots of color-coded LifeAct-EGFP fluorescence intensity in a control cell (left image) and in a cell overexpressing a β-catenin mutant (right image). White arrows indicate the direction of migration; scale bars, 10 μm. The cells are derived from the same sets of cells analysed in Supplementary Fig. 4d, e. **d,** Upper panel: schematics explaining the measurements illustrated below. 0.0 = cell front and 1.0 = cell rear. Upper graph: mean normalized LifeAct-EGFP fluorescence intensity along the cell front-rear axis in control cells (black line) and cells expressing the dominant negative form of β-catenin (green line). Lower graph: lines connect the highest and middle point of the corresponding curves presented in the graph above. n = number of averaged curves with data from 3 independent experiments; mean ± s.e.m.. Data derived from the same sets of cells analysed in Supplementary Fig. 4e. **e,** Mean values of LifeAct-EGFP intensity for the whole cell area. Open circles represent control cells and green squares represent values of PGCs overexpressing the β-catenin mutant. n = number of cells analysed in 3 independent experiments; mean ± s.e.m.; *P* value: two-sided Student's *t*-test. Data derived from the same sets of cells analysed in Supplementary Fig. 4d. For the β-catenin mutant, the ratio of the signal at the cell front divided by that in the middle is 2.22 ± 0.09 as compared with 2.05 ± 0.08 for the control (mean ± s.e.m.). Source data are provided as a Source Data file.

Supplementary Fig. 5

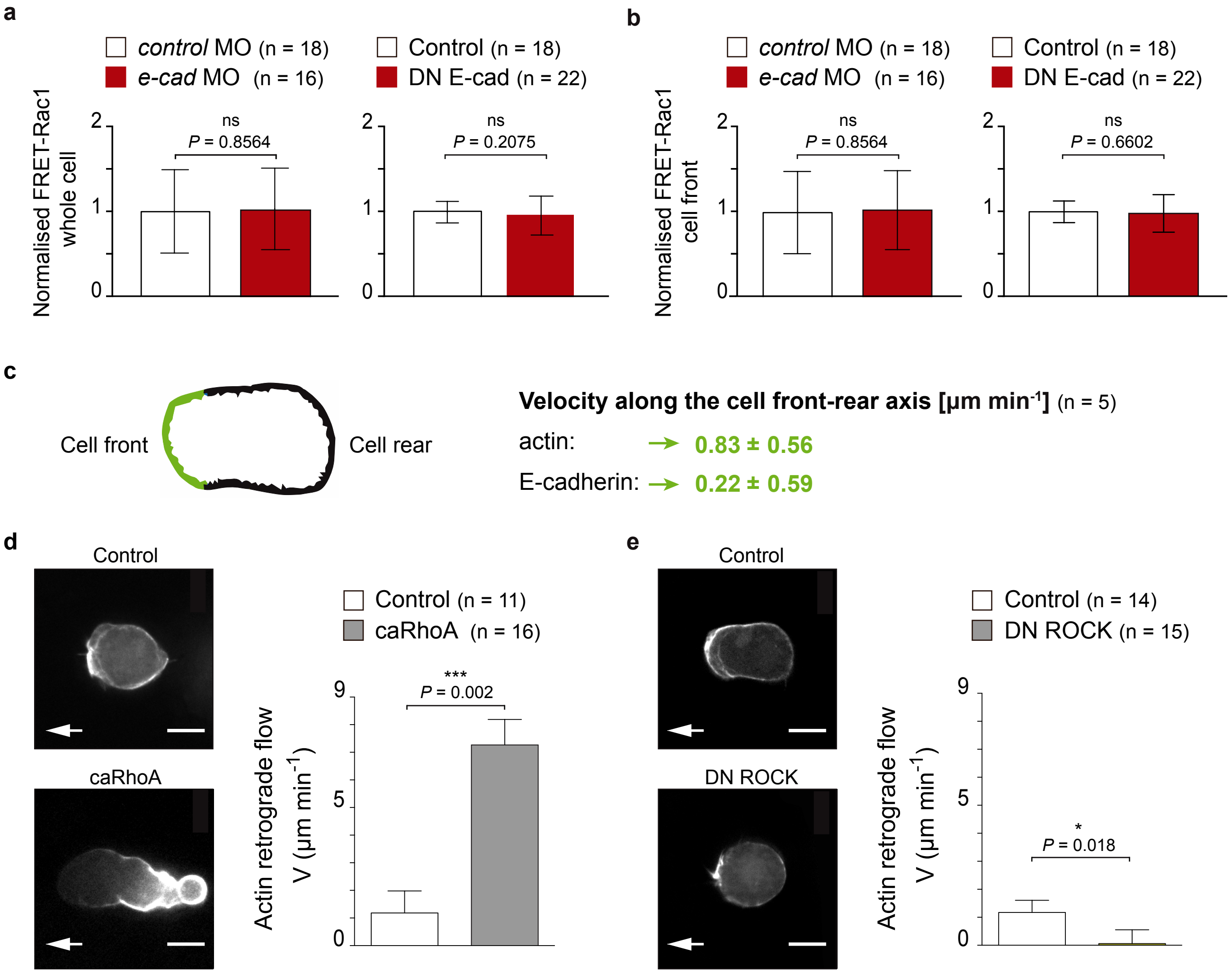

f

Actin velocity along the cell front-rear axis [µm min^{-1}]

	Control (n = 15)	ß-catenin ΔQ28-H134 (n = 15)
1	→ **1.70** ± 0.41	→ **2.90** ± 0.69
2	→ **0.73** ± 0.46	→ **1.56** ± 0.60
3	← **0.01** ± 0.57	← **0.26** ± 0.72
4	← **0.50** ± 0.86	← **1.24** ± 0.79

Supplementary Fig. 5: Regulation of actin polymerization and dynamics by Rac1 activation, contractility and β-catenin.

a, b, Levels of Rac1 activity in the whole cell **(a)** and at the cell front **(b)**, measured using a Rac1 activity FRET reporter. Rac1 activity in PGCs located within embryos treated with anti *e-cadherin* morpholino or expressing the DN E-cadherin was compared to that within control cells (control level was set to 1). n = number of cells from 3 independent experiments; mean ± s.d.; *P* value: calculated using two-sided Student's *t*-test, except for the use of a two-tailed Mann-Whitney *U*-test in the case of the DN E-cad comparison for the whole cell (due to non-normally distributed data). **c,** Simultaneous measurements of actin and E-cadherin flow velocities at the cell front of wild-type PGCs prior to bleb formation (10 seconds measurements in the 25% front part of the cell perimeter, green area). Green arrows indicate the direction of actin flow with respect to the cell front-rear axis. Mean ± s.e.m.; n = number of cells. **d, e,** Snapshots showing the distribution of LifeAct-EGFP in control cells and in PGCs treated with either constitutive active version of RhoA (caRhoA, **d**) or with a dominant negative version of ROCK (DN ROCK, **e**). White arrows indicate the direction of the cell front. Scale bars: 10 μm. Graphs in **d** and **e**: measurements of actin velocity at the cell front performed using the BioFlow software (see Methods) in control cells and in cells overexpressing either caRhoA (**d**) or DN ROCK (**e**). n = number of cells from 3 independent experiments; mean ± s.e.m.; *P* value: two-sided Student's *t*-test. **f,** Measurements of actin velocity along the cell perimeter of PGCs upon β-catenin knockdown conducted as in Fig. 2f. Black and green arrows indicate the direction of actin flow with respect to the cell front-rear axis. Values indicate mean ± s.e.m.; n = number of cells from 3 independent experiments. Source data are provided as a Source Data file.

Supplementary Fig. 6

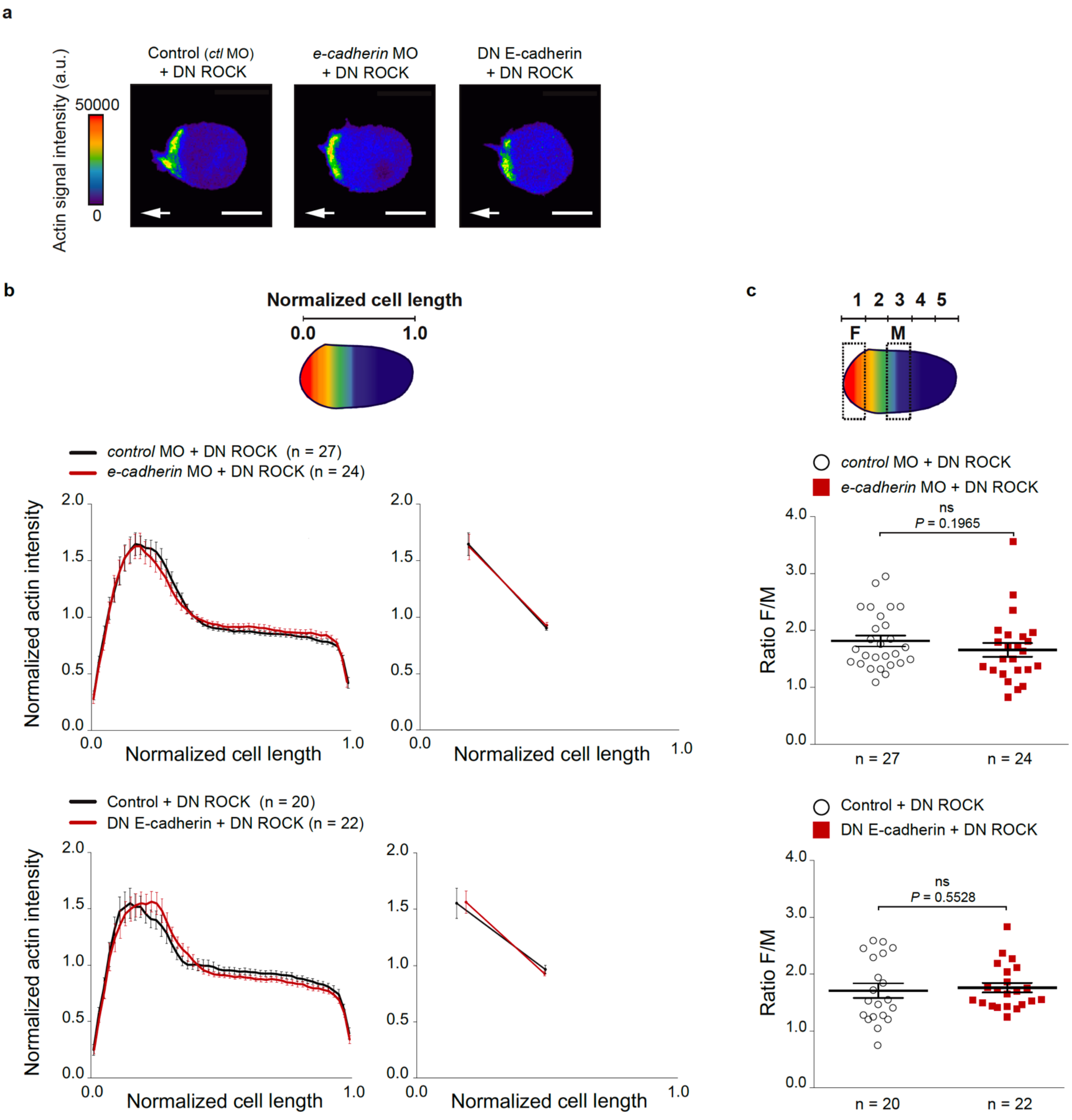

Supplementary Fig. 6: Effect of changes in cortical contractility on actin distribution in PGCs. a, Snapshots of color-coded LifeAct-EGFP fluorescence intensity in cells expressing DN ROCK and treated with *control* MO (left panel), *e-cadherin* MO (middle panel), or DN E-cadherin (right panel). White arrows indicate the direction of migration; scale bars, 10 μm. Cells are derived from the same sets of cells analysed in Supplementary Fig. 6b, c. **b,** Upper panel: schematics explains the measurements illustrated below. 0.0 = cell front and 1.0 = cell rear. Left graphs: mean normalized LifeAct-EGFP fluorescence intensity along the cell front-rear axis in cells expressing DN ROCK together with either a *control* MO or *control* RNA (black lines) or *e-cadherin* MO or DN E-cadherin (red lines). Right graphs: lines connecting the highest and middle point of the corresponding graphs on the left. n = number of averaged curves with data from 4 independent experiments for morpholino and 3 independent experiments for DN E-cadherin; mean ± s.e.m.. Data derived from the same sets of cells analysed in Supplementary Fig. 6c. **c,** Upper schematics: explanation of the measurements presented in the graphs below. Cells were divided into 5 parts and the ratio between the mean value of LifeAct-EGFP signal in segment 1 (F, front) and that in segment 3 (M, middle) was calculated. Graphs: F/M ratios for cells expressing DN ROCK with either *control* MO or *control* RNA (open circles) or cells expressing DN ROCK with *e-cadherin* MO or DN E-cadherin (red squares). n = number of cells from 4 independent experiments for morpholino and 3 independent experiments for DN E-cadherin; mean ± s.e.m.; *P* value: two-tailed Mann-Whitney *U*-test. Data derived from the same sets of cells analysed in Supplementary Fig. 6b. Source data are provided as a Source Data file.

Supplementary Fig. 7

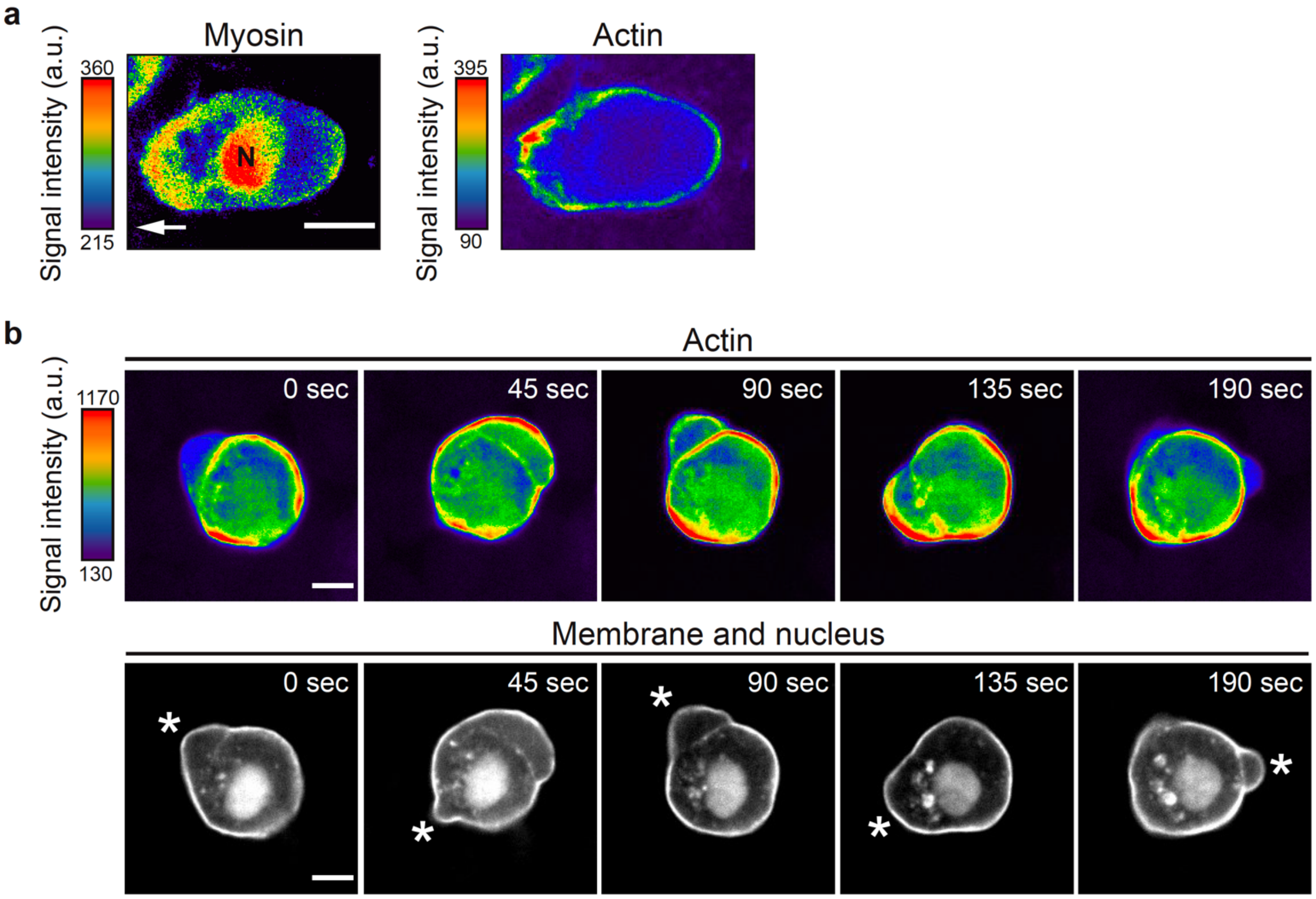

Supplementary Fig. 7: Distribution of actin, myosin, and blebs in polarized and apolar PGCs. a, Snapshots showing the distribution of myosin (Myosin light chain 12.1-EGFP) and actin (LifeAct-mCherry) in a polarized PGC (see Fig 3a for an additional example). The snapshots are derived from Supplementary Movie 4, time point 30 sec. White arrows indicate the direction of migration; N = myosin nuclear localization that might reflect a possible function of the protein in the nucleus (as described for human Myl12a also known as MRLC3[1]); a.u. = arbitrary units; scale bars, 10 µm. The experiment was repeated three times. **b,** Time-lapse images showing the distribution of actin (LifeAct-EGFP, upper panels) and blebs (mCherry-farnesylated, lower panels) in PGCs forced into an apolar state by a uniform high expression of the guidance cue Cxcl12a. A nuclear marker (mScarlet-i-nls) was co-injected with *cxcl12a* mRNA to identify PGCs expressing the chemokine. White stars in the membrane channel indicate the positions where blebs form. a.u. = arbitrary units; scale bars, 10 µm; sec = seconds. Snapshots are derived from the Supplementary Movie 6. The experiment was repeated three times.

Supplementary Fig. 8

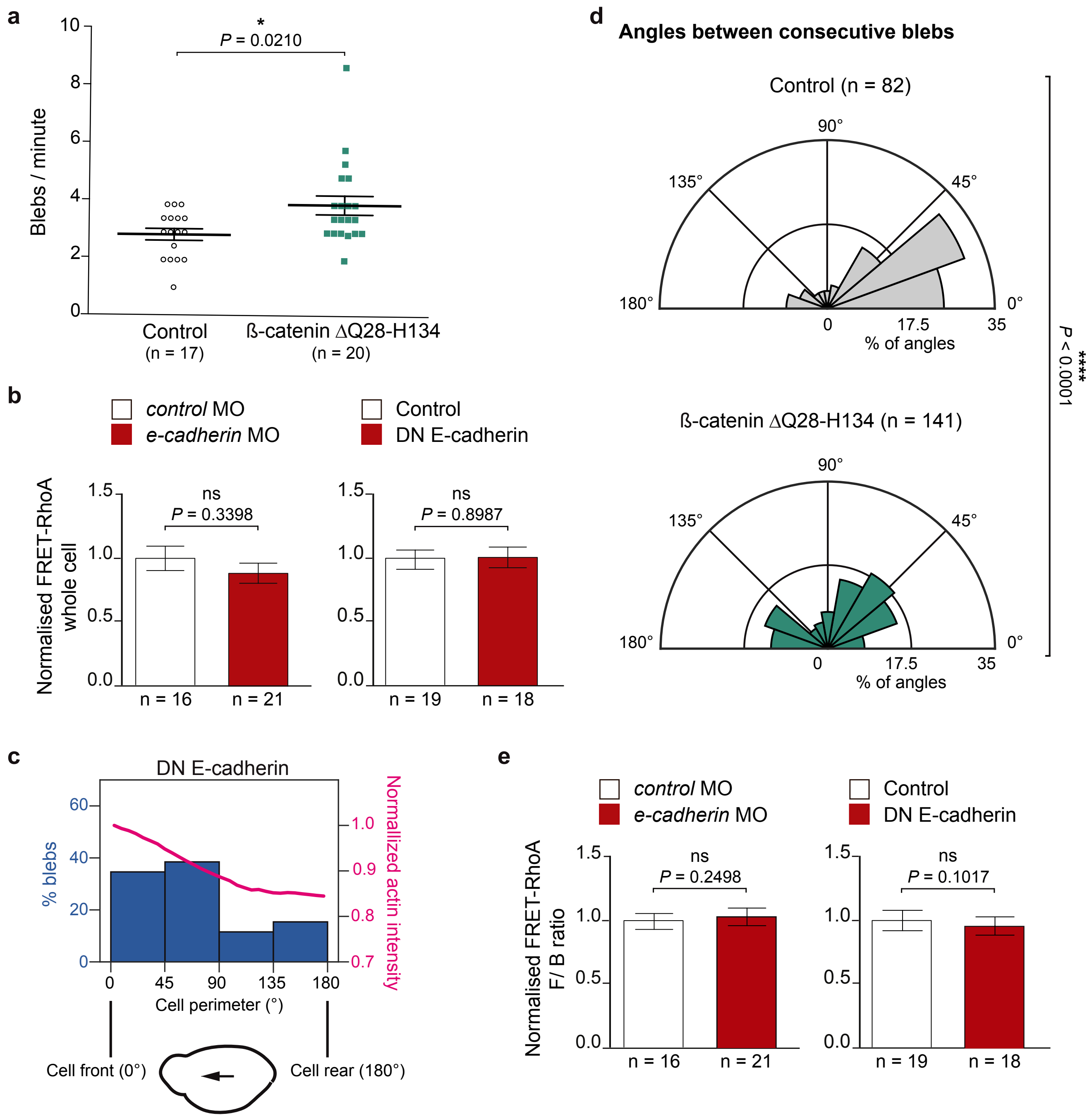

Supplementary Fig. 8: RhoA activity upon E-cadherin knockdown and effects of E-cadherin and β-catenin inhibition on bleb formation in PGCs. a, Frequency of bleb formation in polarized, motile PGCs upon inhibition of β-catenin function. n = number of cells from 3 independent repeats; mean ± s.e.m.; *P* value: two-tailed Mann-Whitney *U*-test. **b,** Levels of RhoA activity in polarized PGCs upon knockdown of E-cadherin using either morpholinos (left graph) or DN E-cadherin overexpression within the PGCs (right graph). FRET-RhoA ratios were normalized relative to the corresponding control values. n = number of PGCs from 3 experimental repeats for *e-cadherin* morpholino and 4 repeats for DN E-cadherin. Data are derived from the same set of cells analysed in Supplementary Fig. 8e. Mean ± s.d.; *P* values: two-tailed Mann-Whitney *U*-test. **c,** Graph: percentage of blebs initiating at different angles around the cell perimeter (left y-axis, blue columns) and normalized actin intensity (right y-axis, magenta line) of cells expressing the DN E-cadherin analysed in a similar way to the control cells presented in Fig 3b). A total number of 4 representative cells and 26 blebs were analysed. **d,** Distribution of angles between consecutive blebs in control cells (grey polar plot) and cells expressing the β-catenin mutant (green polar plot). n = number of angles obtained from 3 independent repeats (17 cells for Control and 20 cells for the expression of the β-catenin mutant); *P* value: two-tailed Kolmogorov-Smirnov test. **e,** Ratios between RhoA activation at the leading edge and at the rear of PGCs located within *e-cadherin* morpholino-treated embryos and in cells expressing the DN E-cadherin form. Controls were set to 1. Data are derived from the same set of cells analysed in Supplementary Fig. 8b. Mean ± s.d.; *P* values: two-sided Student's *t*-test. Source data are provided as a Source Data file.

Supplementary Fig. 9

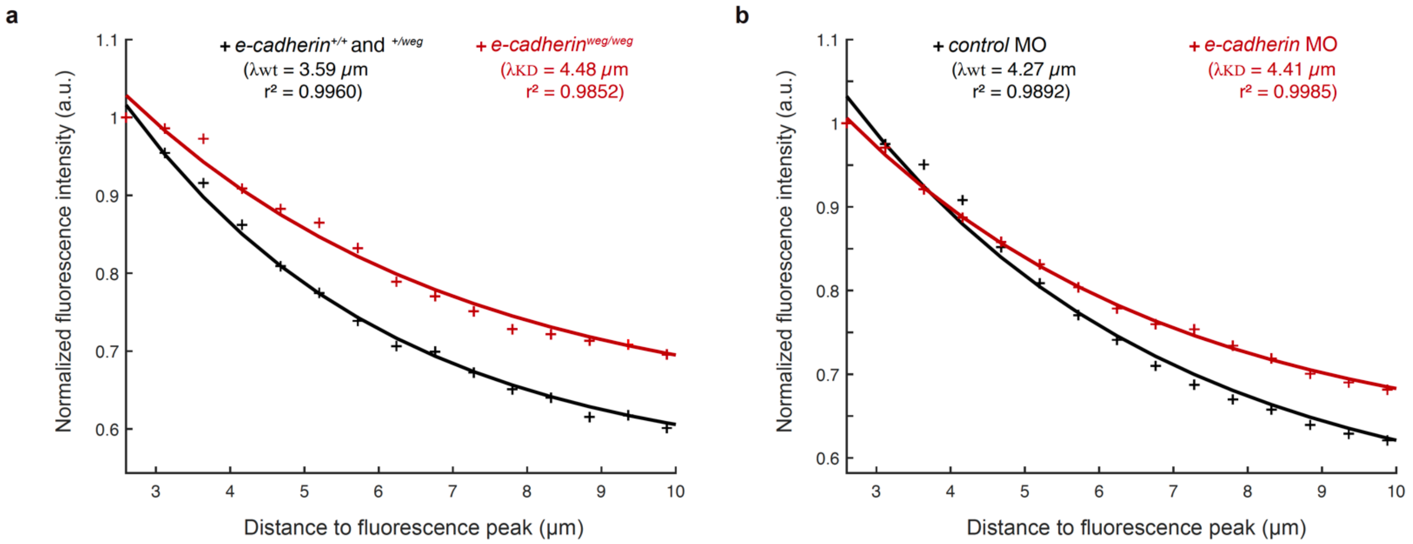

Supplementary Fig. 9: Model of the effect of E-cadherin KD on the distribution of actin in PGCs. a, b, Exponential fits of the actin fluorescence intensity in the PGCs derived from the signal profiles shown in Supplemental Fig. 4a. The effect of decreasing the fraction of actin linked to E-cadherin was examined in *e-cadherin*$^{weg/weg}$ mutant embryos (left, red data points) or in embryos treated with the *e-cadherin* MO (right, red data points). The corresponding data for DN E-cadherin and β-catenin are presented in Fig. 4c. As predicted by the transport decay model, in each case the decay length of the treatment (λ_{KD}) is larger than in the control (λ_{wt}) situation: $\lambda_{wt} < \lambda_{KD}$. This suggests that the overall actin polarity is reduced when the friction with the environment is reduced.

Supplementary Fig. 10

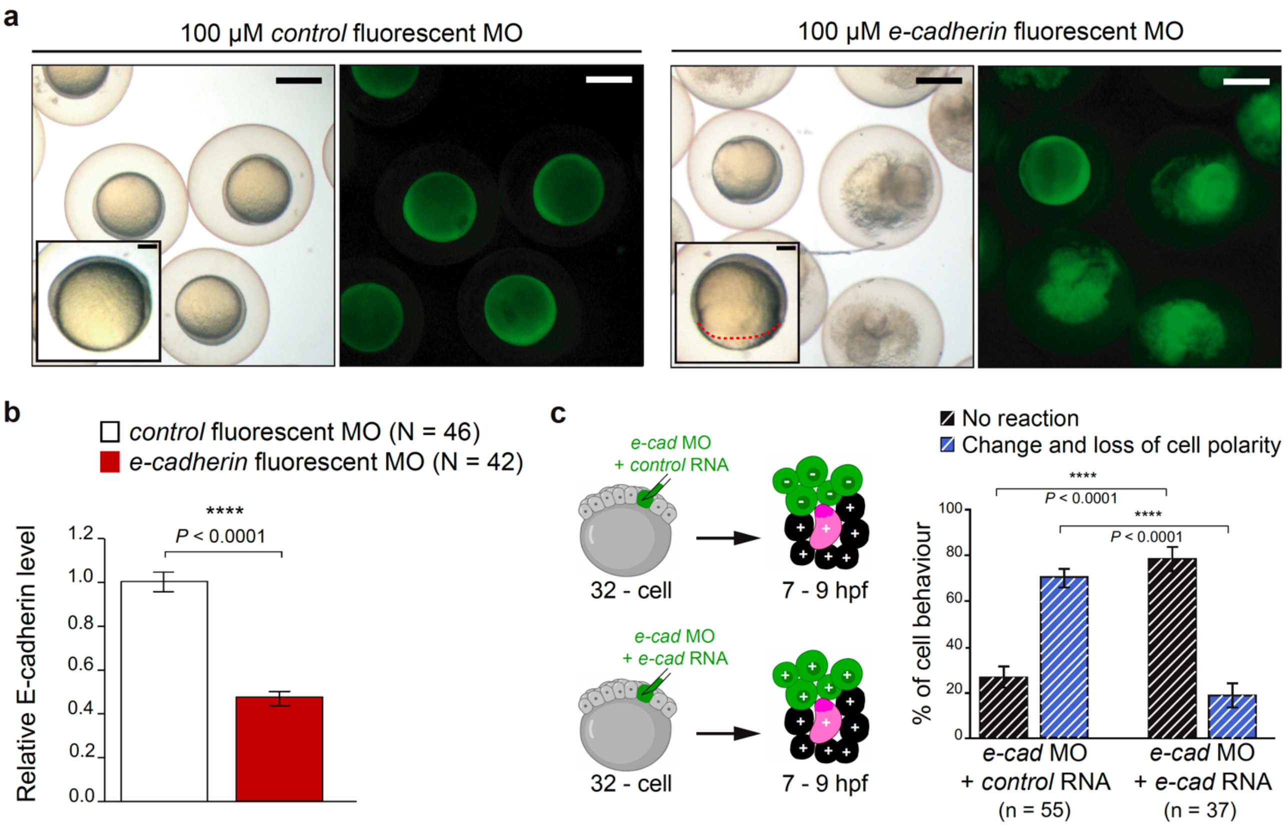

Supplementary Fig. 10: Characterization of the fluorescently labelled *e-cadherin* morpholino. a, Representative images of embryos injected with either fluorescently labelled *control* morpholino (left panels) or labelled *e-cadherin* morpholino (right panels). Embryos were imaged at 10 hpf. Bright-field images, lower magnification; scale bars, 500 µm. Bright-field images, insets; the dotted red line indicates epiboly delay; scale bars, 150 µm. Fluorescent images show the same embryos presented in the lower magnification bright-field panels. The green signal indicates the presence of either *control* (left panel) or *e-cadherin* (right panel) fluorescent morpholino in the injected embryos; scale bars, 500 µm. The experiment was repeated three times. **b,** E-cadherin levels in embryos injected with 100 µM of either *control* or *e-cadherin* fluorescent morpholino, with the level in the control set to 1. Embryos were fixed at 10 hpf and stained using anti-E-cadherin antibody. N = number of embryos from 3 biological replicates. Normalized mean ± s.e.m.; *P* value: two-tailed Mann-Whitney *U*-test. **c,** Schematics illustrating the two different scenarios analysed in the graph. The graph shows the percentage of PGCs presenting no reaction (black striped columns) or change and loss of polarity (blue striped columns) upon contact with a clone co-injected with *e-cadherin* MO and *control* RNA or with *e-cadherin* MO and an *e-cadherin* RNA insensitive to the MO. Mean ± s.e.m.; *P* value: two-tailed Mann-Whitney *U*-test; n = number of contact events from 6 independent experiments. Source data are provided as a Source Data file.

Supplementary File 1A. Experimental Design

Experiment	Fish lines used	Stage of injection	Material injected		Used in Figure
Migration tracks	*weg*^{tx230} mutant	1-cell	*mcherry-h2b-globin3'UTR* mRNA (mCherry labelling nuclei of all cells) +	*lifeact-ypet-nos3'UTR* (YPet labelling filamentous actin of PGCs)	Fig. 1a and Supplementary Fig. 1a
Migration tracks	homozygous *kop:lifeact-egfp-nanos3'UTR* (EGFP labelling filamentous actin of PGCs)	1-cell	*mcherry-h2b-globin3'UTR* mRNA (mCherry labelling nuclei of all cells) +	1. *control* or *e-cadherin* MO<br>2. *pcd14-nos3'UTR* or *zfecdh-W2A-nos3'UTR*	1. Fig. 1b and Supplementary Fig. 1a<br>2. Fig. 1c and Supplementary Fig. 1a
Actin signal intensity profiles	*weg*^{tx230} mutant	1-cell	*lifeact-ypet-nos3'UTR* (YPet labelling filamentous actin of PGCs)	*mcherry-f-nanos3'UTR* (mCherry labelling membrane of PGCs)	Fig. 2b, Supplementary Fig. 4a, b, and Supplementary Fig. 9a
Actin signal intensity profiles	homozygous *kop:lifeact-egfp-nanos3'UTR*	1-cell	*mcherry-f-nanos3'UTR* (mCherry	1. *control* or *e-cadherin* MO	1. Fig. 2a, b, Supplementary Fig. 4a, b and

	(EGFP labelling filamentous actin of PGCs)		labelling membrane of PGCs)	2. *pcd14-nos3'UTR* or *zfecdh-W2A-nos3'UTR*<br>3. *pcd14-nos3'UTR* or *zfctnn1b1Δ28-134-nanos3'UTR*	Supplementary Fig. 9b<br>2. Fig. 2b, Supplementary Fig. 4a, b and Fig. 4c<br>3. Supplementary Fig. 4c, d, e and Fig. 4c
Actin signal intensity profiles	homozygous *kop:lifeact-egfp-nanos3'UTR* (EGFP labelling filamentous actin of PGCs)	1-cell	*mcherry-f-nanos3'UTR* (mCherry labelling membrane of PGCs)	1. *roc c'-nanos3'UTR* + *control* or *e-cadherin* MO<br>*2.* *roc c'-nanos3'UTR* + *pcd14-nos3'UTR* or *zfEcdh-W2A-nos3'UTR*	1. Supplementary Fig. 6<br>2. Supplementary Fig. 6
Actin dynamics, protrusions frequency, and protrusions coordination	*kop:lifeact-egfp-nanos3'UTR* (EGFP labelling filamentous actin of PGCs) and *kop:mcherry-f-nos3'UTR* (mCherry labelling membrane of PGCs)	1-cell	1. *control* or *e-cadherin* MO<br>2. *pcd14-nos3'UTR* or *zfecdh-W2A-nos3'UTR*<br>3. *pcd14-nos3'UTR* or *zfctnn1b1Δ28-134-nanos3'UTR*<br>4. *pcd14-nos3'UTR* or *zrhoAV14-nos3'UTR*<br>5. *pcd14-nos3'UTR* or *roc c'-nanos3'UTR*		1. Fig. 2c, f and 3c, e<br>2. Fig. 2d, f, Fig. 3b, c, f, and Supplementary Fig. 8c<br>3. Supplementary Fig. 5f and Supplementary Fig. 8a, d

					4. Supplementary Fig. 5d<br>5. Supplementary Fig. 5e
Actin and cytosol dynamics	homozygous *kop:lifeact-mcherry-nos3'UTR* (mCherry labelling filamentous actin of PGCs)	1-cell	*egfp-nos3'UTR*		Fig. 2e
Counting % of ectopic PGCs	homozygous *kop:lifeact-egfp-nanos3'UTR* (EGFP labelling filamentous actin of PGCs)	1-cell	*mcherry-h2b-globin3'UTR* mRNA (mCherry labelling nuclei of all cells)	*1. pcd14-nos3'UTR* or *zfecdh-W2A-nos3'UTR*<br>*2. pcd14-nos3'UTR* or *zfctnn1b1Δ28-134-nanos3'UTR*	1. Fig. 1d<br>2. Supplementary Fig. 3
RhoA-FRET	*kop:mcherry-f-nanos3'UTR* (mCherry labelling membrane of PGCs)	1-cell	1. *rhoAFRET ypet-noCT-nos3'UTR + control* or *e-cadherin* MO<br>2. *rhoAFRET ypet-noCT-nos3'UTR + pcd14-nos3'UTR* or *zfecdh-W2A-nos3'UTR*		1. Supplementary Fi. 8b, e<br>2. Supplementary Fig. 8b, e
Morpholino-treated clones	homozygous *kop:lifeact-mcherry-nanos3'UTR*	32-cell	1. *control* or *e-cadherin* fluorescent MO		1. Fig. 5<br>2. Supplementary Fig. 10c

	(mCherry labelling filamentous actin of PGCs)		2. *e-cadherin* fluorescent MO + *pa-gfp-globin3'UTR* or *zfecdh-W2A-globin3'UTR*	
Assessment of the somatic phenotype induced by *e-cadherin* MO and rescue experiments	AB and AB/TL	1-cell	1. *control* or *e-cadherin* MO<br>2. *control* MO + *pa-gfp-globin3'UTR* or *control* MO + *zfecdh-globin3'UTR* or *e-cadherin* MO + *pa-gfp-globin3'UTR* or *e-cadherin* MO + *zfecdh-globin3'UTR*<br>3. *control* or *e-cadherin* fluorescent MO	1. Supplementary Fig. 1c, d<br>2. Supplementary Fig. 1e<br>3. Supplementary Fig. 10a, b
Assessment of the somatic phenotype induced by global expression of DN E-cadherin	AB and AB/TL	1-cell	*pa-gfp-globin3'UTR* or *zfecdh-globin3'UTR*	Supplementary Fig. 1g
Subcellular localization of DN E-cadherin-EGFP	AB	1-cell	1. *mcherry-f-globin3'UTR* (mCherry labelling membrane of all cells) + *zfecdh-W2A-egfp-globin3'UTR*<br>2. *mcherry-f-nanos3'UTR* (mCherry labelling membrane in the PGCs) + *zfecdh-W2A-egfp-nanos3'UTR*	Supplementary Fig. 1f

Rac1-FRET	*kop:mcherry-f-nanos3'UTR* (mCherry labelling membranes of PGCs)	1-cell	1. *racFRET ypet-noCT-nos3'UTR* + *control* or *e-cadherin* MO<br>2. *racFRET ypet-noCT-nos3'UTR* + *pcd14-nos3'UTR* or *zfecdh-W2A-nos3'UTR*	Supplementary Fig. 5a, b
Actin and blebs distribution in apolar PGCs	*kop:lifeact-egfp-nanos3'UTR* (EGFP labelling filamentous actin of PGCs) and *kop:mcherry-f-nos3'UTR* (mCherry labelling membrane of PGCs)	1-cell	*sdf1a-nanos3'UTR* + *mscarlet-i-nls-nanos3'UTR* (mScarlet labelling nucleus of PGCs)	Supplementary Fig. 7b
E-cadherin-EGFP subcellular distribution	*kop:mcherry-f-nanos3'UTR* (mCherry labelling membranes of PGCs)	1-cell	*zfecdh-egfp-nos3'UTR*	Supplementary Fig. 2
E-cadherin and actin dynamics	homozygous *kop:lifeact-mcherry-nanos3'UTR* (mCherry labelling filamentous actin of PGCs)	1-cell	*zfecdh-egfp-nos3'UTR*	Supplementary Fig. 5c

Myosin light chain 12.1-EGFP subcellular localization	homozygous *kop:lifeact-mcherry-nanos3'UTR* (mCherry labelling filamentous actin of PGCs)	1-cell	*myl12.1-egfp-nos3'UTR*	Fig. 3a and Supplementary Fig. 7a

Supplementary File 1B. Constructs Cloned for this Work

Construct (internal number)	F-primer, 5'-3' (internal number)	R-primer, 5'-3' (internal number)	Amount injected
zfecdhW2A-globin3'UTR (D081), expressing DN E-cadherin in all cells	GTGAAGAGAGGAG CTATCATCCCTCCT ATC (F208)	GCGCTTGTTCTTG GTCAGGTCCACG (F209)	As indicated in each experiment
zfecdh-W2A-egfp-globin3'UTR (D079), expressing DN E-cadherin-EGFP in all cells	GTGAAGAGAGGAG CTATCATCCCTCCT ATC (F208)	GCGCTTGTTCTTG GTCAGGTCCACG (F209)	400 pg (210 nM)
zfecdh-W2A-nos3'UTR (D095), expressing DN E-cadherin in the PGCs	GTGAAGAGAGGAG CTATCATCCCTCCT ATC (F208)	GCGCTTGTTCTTG GTCAGGTCCACG (F209)	400 pg (210 nM)
zfecd-W2A-egfp-nos3'UTR (D082), expressing DN E-cadherin-EGFP in the PGCs	GTGAAGAGAGGAG CTATCATCCCTCCT ATC (F208)	GCGCTTGTTCTTG GTCAGGTCCACG (F209)	400 pg (210 nM)
zfecdh-globin3'UTR (C264), expressing E-cadherin in all cells	GATCTCGAGCTCAA GCTTCGAATTCT (E206)	TTAGTCCTCTCCG CCACCGTACATAT (E207)	300 pg (160 nM) (or as indicated for

			Supplementary Fig. 1e)
zfctnn1b1Δ28-134-nanos3'UTR (D606), expressing β-catenin mutant in the PGCs	GTGGTCAACCTCATCAACTACCAGG (G557)	GTGGTCAACCTCATCAACTACCAGG (G558)	300 pg (160 nM)
lifeact-mcherry-nos3'UTR (D554), expressing LifeAct-mCherry in the PGCs	CATCTCAAAGGAAGAAGGTGCTGGTGCTGGTGCTGGTGCTATGGTGAGCAAGGGCGAGGAG (G183)	CTTTCGAATTTCTTGATCAAATCTGCGACACCCATGTTTGCCCCGGGCTGCAGGAATTC (G184)	200 pg (195 nM)
lifeact-ypet-nos3'UTR (C808), expressing LifeAct-YPet in the PGCs	AGAAATTCGAAAGCATCTCAAAGGAAGAAGGGGATCCACCGGTCGCCACC (E856)	AATGTCCGCTCTCGACGTCGAGATGGCCGCTTTACTTATAGAGCTCGTTC (E857)	200 pg (195 nM)
zfecdh-egfp-nos3'UTR (C497), expressing E-cadherin-EGFP in the PGCs			200 pg (105 nM)
mscarlet-i-nls-nos3'UTR (E122), expressing mScarlet in the nucleus of the PGCs. The pmScarlet-i fluorophore was a gift from Dorus Gadella (Addgene plasmid #85044; http://n2t.net/addgene:85044)[2]	CTTAGATCTGAATTCCTGCAACCATGGTGAGCAAGGGCGA (H718)	ACCTTTCTCTTCTTTTTTGGCTTGTACAGCTCGTCCATGCC (H719)	140 pg (160 nM)
*myl12.1-egfp-nos3'UTR (*D359), expressing Myosin light chain 12.1-EGFP in the PGCs	GGAAGATCTACCATGTCGAGCAAACGCGCCAAGGGA (F757)	CGGGGTACCCCGCCATCGTCTTTGTCTTTGGCTCCGTGTTTCA (F758)	200 pg (225 nM)

Supplementary File 1C. Additional Constructs Used in this Work

Construct (internal number)	Amount injected
pcd14-nos3’UTR (554)[3], used as injection control RNA	Respective to the experimental RNA
pa-gfp-globin3’UTR (A918)[4], used as injection control RNA	Respective to the experimental RNA
zfrhoAV14-nos3'UTR (B282)[5], expressing the constitutively active version of RhoA in the PGCs	40 pg<br>(50 nM)
roc c’-nos3’UTR (432)[6], expressing the DN ROCK in the PGCs	360 pg<br>(230 nM)
mcherry-h2b-globin3’UTR (B325)[3], expressing mCherry-H2B in all cells	100 pg<br>(120 nM)
mcherry-f-globin3’UTR (A709)[7], expressing farnesylated mCherry in all cells	100 pg<br>(145 nM)
mcherry-f-nanos3’UTR (A906)[4], expressing farnesylated mCherry in the PGCs	100 pg<br>(105 nM)
rhoAFRET ypet-noCT-nos3’UTR (A676)[5], expressing the RhoA FRET biosensor in the PGCs	240 pg<br>(120 nM)
egfp-nos3'UTR (355)[8], expressing cytoplasmic EGFP in the PGCs	100 pg<br>(105 nM)
racFRET ypet-noCT-nos3’UTR (A422)[5], expressing the Rac1 FRET biosensor in the PGCs	240 pg<br>(125 nM)
sdf1a-nos3’UTR (640), expressing the chemokine Cxcl12a in the PGCs	200 pg<br>(325 nM)

Supplementary File 1D. Morpholino Antisense Oligonucleotides Used in this Work

Morpholino (internal number)	Sequence 5'-3'	Concentration
e-cadherin (78)	ATCCCACAGTTGTTACACAAGCCAT	100 μM (except for Supplemental Fig. 1c)
control (1)	CCTCTTACCTCAGTTACAATTTATA	Respective to the experimental MO

Supplementary References:

1. Höpker, K. *et al.* AATF/Che-1 acts as a phosphorylation-dependent molecular modulator to repress p53-driven apoptosis. *EMBO J.* **31**, 3961–3975 (2012).
2. Bindels, D. S. *et al.* mScarlet: a bright monomeric red fluorescent protein for cellular imaging. *Nat. Methods* **14**, 53–56 (2017).
3. Goudarzi, M. *et al.* Identification and Regulation of a Molecular Module for Bleb-Based Cell Motility. *Dev. Cell* **23**, 210–218 (2012).
4. Meyen, D. *et al.* Dynamic filopodia are required for chemokine-dependent intracellular polarization during guided cell migration in vivo. *Elife* **4**, (2015).
5. Kardash, E. *et al.* A role for Rho GTPases and cell-cell adhesion in single-cell motility in vivo. *Nat. Cell Biol.* **12**, 47–53; sup pp 1-11 (2010).
6. Blaser, H. *et al.* Migration of Zebrafish Primordial Germ Cells: A Role for Myosin Contraction and Cytoplasmic Flow. *Dev. Cell* **11**, 613–627 (2006).
7. Mahabaleshwar, H., Tarbashevich, K., Nowak, M., Brand, M. & Raz, E. -arrestin control of late endosomal sorting facilitates decoy receptor function and chemokine gradient formation. *Development* **139**, 2897–2902 (2012).
8. Doitsidou, M. *et al.* Guidance of Primordial Germ Cell Migration by the Chemokine SDF-1. *Cell* **111**, 647–659 (2002).
