# Supplementary material for: E-cadherin focuses protrusion formation at the front of migrating cells by impeding actin flow
Source: Nat Commun. 2020 Oct 26;11:5397. doi: 10.1038/s41467-020-19114-z (PMC7588466; doi:10.1038/s41467-020-19114-z)
Supplement: Supplementary file 2 — Description of Additional Supplementary Files [file 41467_2020_19114_MOESM2_ESM.pdf]

Description of Additional Supplementary Files

File Name: Supplementary Movie 1

Description: **Actin distribution and dynamics in migrating PGCs.** Time-lapse movies of polarized, migrating PGCs expressing LifeAct-EGFP to visualize the actin network and the actin-rich structures at the leading edge (left side). Embryos were treated with either control MO or e-cadherin MO (first two movie segments) or their PGCs expressed Control RNA, or RNA encoding for DN E-cadherin (last two movie segments). The movies show the same exemplary cells presented in Fig. 2c, d. sec = seconds.

File Name: Supplementary Movie 2

Description: **Cytoplasm and actin flows in polarised PGCs.** Time-lapse movie of a polarised PGC in which simultaneous analysis of the cytoplasm and actin flow was conducted using the BioFlow software. The black arrow indicates the direction of migration. For both channels, the arrows indicating the flow are placed on top of the cell body shape obtained by edge detection for the cytoplasmic signal (grey background). Sec = seconds. Color-coded arrows: Cytoplasmic EGFP, blue = 0.0 µm min-1 and red = 44.4 µm min-1 ; LifeAct-mCherry, blue = 0.0 µm min-1 and red = 46.8 µm min-1 . Black stars in the LifeAct-mCherry channel point at positions where blebs that are initially actin-free can be observed.

File Name: Supplementary Movie 3

Description: **Measurements of actin flow along the cell perimeter.** Time-lapse movies of polarised PGCs in which analysis of the actin flow was conducted using the BioFlow software as described in the first introductory part of the movie. Embryos were treated with either control MO or e-cadherin MO, while in the last movie segment PGCs expressing Control RNA or RNA encoding for DN E-cadherin are presented. Color-coded arrows representing the flow are placed on top of the cell body shape obtained by edge detection for the actin signal (grey background). Black arrows indicate the direction of migration; sec = seconds

File Name: Supplementary Movie 4

Description: **Myosin and actin distribution in polarised PGCs.** Time-lapse movies showing the distribution of Myosin light chain 12.1-EGFP and LifeAct-mCherry in two exemplary, polarized PGCs. The same cells are presented in Fig. 3a and Supplementary Fig. 7a. White arrows indicate the direction of migration; sec = seconds. Color-coded signal intensities in arbitrary units: Cell 1 Myl12.1-EGFP, blue = 230 and red = 400; Cell 1 LifeAct-mCherry, blue = 70 and red = 560; Cell 2 Myl12.1-EGFP, blue = 215 and red = 360; Cell 2 LifeAct-mCherry, blue = 90 and red = 395.

File Name: Supplementary Movie 5

Description: **Actin and blebs distribution in apolar PGCs expressing Cxcl12a.** Time-lapse movie showing the distribution of actin and blebs in PGCs forced into an apolar state by a uniform high expression of the guidance cue Cxcl12a. A nuclear marker was co-injected with cxcl12a mRNA to identify PGCs expressing the chemokine. sec = seconds.

File Name: Supplementary Movie 6

Description: **Cell shape and actin localisation in migrating PGCs.** Time-lapse movies of polarized, migrating PGCs expressing LifeAct-EGFP and farnesylated mCherry (to label the cell membrane). Embryos were treated with either control MO or e-cadherin MO or their PGCs expressed DN E-cadherin as indicated in the movie. sec = seconds.

File Name: Supplementary Movie 7

Description: ***'No reaction'* behaviour of a PGC upon contact with MO-treated cells.** Time-lapse movie showing an example of no reaction of a polarised PGC (Cell of interest) contacts fluorescently labelled MO-treated cells (green cells; e-cadherin MO in this specific example). The dark area is occupied by unlabelled, wild-type somatic cells. LifeAct in the PGC is marked in magenta and the brighter region corresponds to the cell front. The yellow arrow appears at the time the PGC interacts with the green cells and points at the position where the contact takes place. Upon contact, the enrichment of actin is unaltered and the PGC continues to migrate in the same direction. sec = seconds.

File Name: Supplementary Movie 8

Description**: *'Change of polarity'* behaviour of a PGC upon contact with MOtreated cells.** Example of change of polarity of a polarised PGC (Cell of interest) contacts fluorescently labelled MO-treated cells (green cells; e-cadherin MO in this specific example). The dark area is occupied by unlabelled, wild-type somatic cells. LifeAct in the PGC is marked in magenta and the brighter region corresponds to the cell front. The yellow arrow appears at the time the PGC interacts with the green cells and points at the position where the contact takes place. Upon contact, actin enrichment shifts towards one side of the cell and a new leading edge is generated. sec = seconds.

File Name: Supplementary Movie 9

Description**: *'Loss of polarity'* behaviour of a PGC upon contact with MO-treated cells.** Time-lapse movie of a polarised PGC (Cell of interest) losing polarity (loss of polarity) upon contact with fluorescently labelled MO-treated cells (green cells; e-cadherin MO in this specific example). The dark area is occupied by unlabelled, wild-type somatic cells. LifeAct in the PGC is marked in magenta and the brighter region corresponds to the cell front. The yellow arrow appears at the time the PGC interacts with the green cells and points at the position where the contact takes place. Upon contact, the actin enrichment disappears, and the cell stops moving. sec = seconds.
