# Supplementary material for: E-cadherin focuses protrusion formation at the front of migrating cells by impeding actin flow
Source: Nat Commun. 2020 Oct 26;11:5397. doi: 10.1038/s41467-020-19114-z (PMC7588466; doi:10.1038/s41467-020-19114-z)
Supplement: Supplementary file 12 — Reporting Summary [file 41467_2020_19114_MOESM12_ESM.pdf]

nature research

Corresponding author(s): Erez Raz

Last updated by author(s): Sep 10, 2020

Reporting Summary

Nature Research wishes to improve the reproducibility of the work that we publish. This form provides structure for consistency and transparency in reporting. For further information on Nature Research policies, see Authors & Referees and the Editorial Policy Checklist .

Statistics

For all statistical analyses, confirm that the following items are present in the figure legend, table legend, main text, or Methods section.

n/a	Confirmed	
☐	☒	The exact sample size (*n*) for each experimental group/condition, given as a discrete number and unit of measurement
☐	☒	A statement on whether measurements were taken from distinct samples or whether the same sample was measured repeatedly
☐	☒	The statistical test(s) used AND whether they are one- or two-sided<br>*Only common tests should be described solely by name; describe more complex techniques in the Methods section.*
☒	☐	A description of all covariates tested
☐	☒	A description of any assumptions or corrections, such as tests of normality and adjustment for multiple comparisons
☐	☒	A full description of the statistical parameters including central tendency (e.g. means) or other basic estimates (e.g. regression coefficient) AND variation (e.g. standard deviation) or associated estimates of uncertainty (e.g. confidence intervals)
☐	☒	For null hypothesis testing, the test statistic (e.g. *F*, *t*, *r*) with confidence intervals, effect sizes, degrees of freedom and *P* value noted<br>*Give P values as exact values whenever suitable.*
☒	☐	For Bayesian analysis, information on the choice of priors and Markov chain Monte Carlo settings
☒	☐	For hierarchical and complex designs, identification of the appropriate level for tests and full reporting of outcomes
☒	☐	Estimates of effect sizes (e.g. Cohen's *d*, Pearson's *r*), indicating how they were calculated

Our web collection on statistics for biologists contains articles on many of the points above.

Software and code

Policy information about availability of computer code

Data collection	VisiView software (versions 2.1.4 and 4.2.0.2, Visitron Systems, GmbH)<br>VisiView software (version 4.0.0.14, Visitron Systems, GmbH)<br>ZEN software (version lite 2012, Zeiss, Germany)<br>ZEN software (version 2010 B SP1, Zeiss, Germany)<br>ZEN software (version 2014 SPI, Zeiss, Germany)
Data analysis	Vector NTI (Invitrogen) version 11.5 and VNTI Explorer<br>MATLAB R2016a and R2018a<br>Python 3.7<br>Fiji/ImageJ 2.0.2-rc-54/1.52p and 2.0.0-rc-69/1.52i<br>MetaMorph (Molecular Devices, LLC, version 7.7.9.0 2012)<br>Imaris Bitplane versions 8 and 9<br>Microsoft Excel versions 14.4.4 and 16.16.3<br>Prism Graph Pad version 6.0d<br>Free open-source Bioflow plug-in Version 2.1.0.1 in free open-source Icy software Version 1.9.8.0.<br>Custom Python script using the following libraries: FEniCS and mshr 2017.2.0, CGAL 4.12, SciPy 1.1.0, NumPy 1.13.3.<br>Custom Python script using the following libraries: Numpy 1.19.0, SciPy 1.5.0, Opencv2

For manuscripts utilizing custom algorithms or software that are central to the research but not yet described in published literature, software must be made available to editors/reviewers. We strongly encourage code deposition in a community repository (e.g. GitHub). See the Nature Research guidelines for submitting code & software for further information.

Data

Policy information about availability of data

All manuscripts must include a data availability statement. This statement should provide the following information, where applicable:

- Accession codes, unique identifiers, or web links for publicly available datasets
- A list of figures that have associated raw data
- A description of any restrictions on data availability

The data that support the findings of this study are available from the corresponding author upon request.

Field-specific reporting

Please select the one below that is the best fit for your research. If you are not sure, read the appropriate sections before making your selection.

[x] Life sciences [ ] Behavioural & social sciences [ ] Ecological, evolutionary & environmental sciences

For a reference copy of the document with all sections, see nature.com/documents/nr-reporting-summary-flat.pdf

Life sciences study design

All studies must disclose on these points even when the disclosure is negative.

Sample size	The sample size was determined based on the experience in the lab and depended on the nature of data acquisition such as the imaging constrains, age of embryos, stages of cell migration and the need to include events that are very infrequent.
Data exclusions	Data was excluded only when the cells lost their polarity (also due to cell division), migrated out of focus and when signal level was too low for conducting intensity-based analysis. In addition, as indicated in the methods time points when blebs formed were excluded for certain types of analysis (e.g. evaluations of actin polarity).
Replication	All of the experiments were repeated at least three times and the results pointed in the same direction.
Randomization	The source of the organisms was always composed of one pool of embryos that was then divided into the different experimental groups.
Blinding	For each of the experiments the embryos were all derived from the same pool and were imaged and analyzed in parallel (controls and experiments). The measurements yielded values that were subjected to statistical analysis to determine if there are differences among treatments.

Reporting for specific materials, systems and methods

We require information from authors about some types of materials, experimental systems and methods used in many studies. Here, indicate whether each material, system or method listed is relevant to your study. If you are not sure if a list item applies to your research, read the appropriate section before selecting a response.

Materials & experimental systems

n/a	Involved in the study	
[ ]	[x]	Antibodies
[x]	[ ]	Eukaryotic cell lines
[x]	[ ]	Palaeontology
[ ]	[x]	Animals and other organisms
[x]	[ ]	Human research participants
[x]	[ ]	Clinical data

Methods

n/a	Involved in the study	
[x]	[ ]	ChIP-seq
[x]	[ ]	Flow cytometry
[x]	[ ]	MRI-based neuroimaging

Antibodies

Antibodies used	Purified Mouse Anti-E-Cadherin, BD Transduction Laboratories, Cat: 610181, Lot: 3290561, Cat: 610182, Lot: 7292525
Validation	This signal provided by the antibody is reduced by lowering the level of E-Cadherin (this work and PNAS August 5, 2014 111 (31) 11389-11394) and is validated for immunohistochemistry based on the supplier information.

Animals and other organisms

Policy information about studies involving animals; ARRIVE guidelines recommended for reporting animal research

Laboratory animals	Zebrafish embryos were used up to the age of 22 hours post fertilization. The embryos were obtained using natural crosses of adult fish.
Wild animals	*Provide details on animals observed in or captured in the field; report species, sex and age where possible. Describe how animals were caught and transported and what happened to captive animals after the study (if killed, explain why and describe method; if released, say where and when) OR state that the study did not involve wild animals.*
Field-collected samples	*For laboratory work with field-collected samples, describe all relevant parameters such as housing, maintenance, temperature, photoperiod and end-of-experiment protocol OR state that the study did not involve samples collected from the field.*
Ethics oversight	The ethical guidelines for maintaining the adult zebrafish are provided and supervised by the city of Muenster and the authorities of the state of Nordrhein-Westfalen.

Note that full information on the approval of the study protocol must also be provided in the manuscript.
